# Tobacco-related neoplasms: survival analysis and risk of death of population data from Florianópolis, SC

**DOI:** 10.11606/s1518-8787.2022056003651

**Published:** 2022-03-29

**Authors:** Ione Jayce Ceola Schneider, Tauana Prestes Schmidt, Vanessa Pereira Correa, Ana Maria Martins dos Santos, Bruna Vanti da Rocha, Leandro Pereira Garcia, Roger Flores Ceccon

**Affiliations:** I Universidade Federal de Santa Catarina Departamento de Ciências da Saúde Araranguá SC Brasil Universidade Federal de Santa Catarina. Departamento de Ciências da Saúde. Araranguá, SC, Brasil; II Universidade Federal de Santa Catarina Programa de Pós-Graduação em Ciências da Reabilitação Araranguá SC Brasil Universidade Federal de Santa Catarina. Programa de Pós-Graduação em Ciências da Reabilitação. Araranguá, SC, Brasil; III Universidade Federal de Santa Catarina Programa de Pós-Graduação em Saúde Coletiva Florianópolis SC Brasil Universidade Federal de Santa Catarina. Programa de Pós-Graduação em Saúde Coletiva. Florianópolis, SC, Brasil; IV Secretaria Municipal de Saúde de Florianópolis Florianópolis SC Brasil Secretaria Municipal de Saúde de Florianópolis. Florianópolis, SC, Brasil

**Keywords:** Tobacco Use Disorder, Neoplasms, epidemiology, Survival Analysis, Risk Factors, Mortality, Proportional Hazards Models

## Abstract

**OBJECTIVE:**

To estimate the probability of survival and prognostic factors for tobacco-related neoplasms in a population-based cohort.

**METHODS:**

This is a cohort with data from the Population-Based Cancer Registry of Florianópolis, southern Brazil, from 2008 to 2012. The Stata 16.0 software was used to estimate the probabilities of survival in five years after diagnosis, by the Kaplan Meier method, and the risk of death, by the Cox regression.

**RESULTS:**

A total of 2,829 cancer records related to smoking were included, more prevalent among males, over 70 years of age, nine years or more of schooling, white, with a partner and metastatic diagnosis. The most frequent groupings were colon and rectum (28.7%), trachea, bronchi and lungs (18.6%) and stomach (11.8%). At follow-up, 1,450 died. Pancreatic cancer had the worst probability of survival (14.3%), followed by liver cancer (19.4%).

**CONCLUSION:**

Risk factors for death and survival rates differ across the 13 types of tobacco-related cancers. Early diagnosis and primary prevention are strategies that must be improved to improve survival and decrease the burden related to these types of cancer.

## INTRODUCTION

Smoking is characterized as an important public health problem and is considered the second most prevalent modifiable risk factor for chronic non-communicable diseases (NCDs) and for global mortality. In Brazil, currently, there are more than 7 million active smokers and 1.2 million passive smokers. Furthermore, 428 people die daily as a result of nicotine dependence, 56.9 billion reais are spent annually on medical expenses and lost productivity, and 156,216 annual deaths could be avoided by reducing tobacco use^1^.

The incidence of tobacco-related cancer has increased significantly in recent years, mainly tumors in the oral cavity, pharynx, esophagus, stomach, colon, rectum, liver, pancreas, larynx, lung, bronchi, trachea, kidneys, urinary bladder, and uterine colon, in addition to acute myeloid leukemia (AML)^2^. In 2015, cancer was one of the main causes of years of life lost due to disability attributable to smoking and was the cause of 20% of all deaths from the disease^3^.

Worldwide, it is estimated that 18 million people were diagnosed with cancer and 9.6 million died in 2018, most of them male and in low- and middle-income countries^4^. In Brazil, the incidence is over 500,000 new cases, mortality exceeded 100,000 deaths in the same period and the expected survival rate for all cancers is approximately 50% in five years^4,5^.

Survival is defined as the period for which a patient remains alive after diagnosis of the disease or treatment initiation. Although it is an important indicator to measure the magnitude of smoking and cancer in the population, survival after the diagnosis of tumors caused by tobacco is still unknown in Brazil. Furthermore, the analysis of survival makes it possible to evaluate the actions in the oncology area implemented in the country in recent years, as well as their prognostic factors. Furthermore, the study of survival allows to know the behavior of the disease, enabling an approach that provides better quality of life to affected individuals^6^.

There are few reports in the scientific literature about the survival of people with tobacco-related cancer, although it is clear that greater cigarette consumption is associated with worse disease progression^2^.

Thus, the aim of this study was to estimate the probability of survival and prognostic factors for smoking-related neoplasms in a population-based cohort.

## METHODS

This is a population-based cohort with data from the Municipal Cancer Registry of Florianópolis, a municipality located in the southern region of the country. It is the capital of the state of Santa Catarina, has approximately 500 thousand residents, and is characterized by its very high Human Development Index (HDI): 0.815, mainly ascribed to longevity and income^7^. The municipality has high coverage and consolidated experience in primary care, reflecting good results in tracking and early detection of the health conditions of its population. In addition, care for oncology patients, in medium and high complexity, is covered by more than six hospital centers, in addition to associations dedicated to raising awareness and seeking the rights of people with cancer.

The participants is composed of individuals registered in the Population-Based Cancer Registry (RCBP) of Florianópolis, from January 1, 2008 to December 31, 2012, with a diagnosis of smoking-related cancer. The cancers included were those with the International Classification of Diseases for Oncology (ICD-O)^8^code considered smoking-related^9^. These include cancers of the lips and oral cavity (C00-C08); nasopharynx (C11); other pharynxes (C09-C10, C12-C14); esophagus (C15); stomach (C16); colon and rectum (C18-C21); liver (C22); pancreas (C25); larynx (C32); trachea, bronchi and lungs (C33-C34); kidney (C64-C66); bladder (C67) and acute myeloid leukemia (C92.0). Cases that had the means of diagnosis registered as not available or in which the diagnosis was exclusively performed by the death verification service were excluded. The cases of cervical and ovarian cancer, despite being tobacco-related, were not included in the analyses, even with tobacco as a causal factor. Cervical mucus from smokers is more mutagenic and suggests an association with DNA damage in women with cervical cancer. In ovarian cancer, the data suggest that it is associated only with the mucinous type, in which smoking may contribute to a faster progression from the benign to the malignant form. These last two were not added to the analyses because they refer exclusively to females and Brazil has specific public policies for women’s health, especially with a strategic action to track cervical cancer.

To make it possible to follow up on cases registered in the RCBP and estimate survival, information regarding deaths, such as date and underlying cause, is needed. For this, the Brazilian Mortality Information System (SIM) of the Ministry of Health was used, with information referring to the municipality of Florianópolis in the period from January 1, 2008 to December 31, 2017. The creation of the database was based on the technique of probabilistic relationship of records, which allows the integration of databases of different nature, contributing to the improvement of the quality of recorded data^12^. For this, the OpenRecLink 3.1 software was used. For data pairing, the following SIM variables were used: death certificate number, name, date of birth, mother’s name, date of death, underlying cause of death; and from the RCBP bank: registration number, name, date of birth, mother’s name. The relationship parameters followed the software instructions. After linking the databases, a single database was created containing all the information relevant to the study, allowing data analysis.

Survival time was calculated as the time, in months, between the date of diagnosis and the date of death or end of follow-up. The maximum follow-up time was 60 months. All cases not found in the SIM were considered alive at the end of follow-up and censored.

The independent variables were gender (female, male), age group (with cutoff points for each type of cancer), race/color (white, black/brown/indigenous/yellow, no information), schooling (9 years or more, 8 years or less, no information), marital status (with partner, no partner, no information), extension of cancer (localized, metastasis, no information) and year of diagnosis (2008, 2009, 2010, 2011, 2012).

Initially, a descriptive analysis was performed with absolute and relative frequency of independent variables. Afterwards, a bivariate analysis was performed between the independent variables and the status variable (censorship/death) to estimate the incidence of deaths in each category and Pearson’s chi-square test was used to test the association between the variables. These analyses were performed for all cases and for each type of cancer.

For each type of cancer, the probability of survival was estimated using the Kaplan-Meier method. We used the log-rank test to compare survival curves stratified by independent variables. The effect of the independent variables was estimated by the semiparametric model of proportional hazards of crude and adjusted Cox. The adjusted analysis included variables with p-value < 0.20. For data analysis and construction of survival graphs, we used the Stata statistical program (Stata Corp, v.16.0. Stata Statistical Software. College Station: StataCorp LP, 2021).

This research was based on ethical principles, based on Resolution No. 466, of December 12, 2012, of the National Health Council, and approved under CAAE: 53518116.1.0000.0121.

This work is the result of the project financed by *Fundação de Amparo à Pesquisa e Inovação* of the State of Santa Catarina, in the *Programa de Pesquisa para o SUS: Gestão Compartilhada em Saúde* (PPSUS – Research Program for the SUS: Shared Management in Health) (Grant No. 2016RT2206).

## RESULTS

### General description

In the period from January 1, 2008 to December 31, 2012, 3,012 cases of tobacco-related cancer were registered by the RCBP in the municipality of Florianópolis. Of these, 189 were excluded because they were diagnosed by death verification service or because the diagnostic method was not available. Thus, the population of this study is composed of 2,829 records (Table 1).

**Table 1:** Description and survival of registered cases of tobacco-related cancer, Florianópolis, 2008–2017.

Variables	n (%)	Deaths	p^a^	S(t) (95%CI)	p^b^

		n (%)			
Sex			0.009		
Female	1,104 (39.0)	532 (48.2)		51.8 (48.8–54.7)	
Male	1,725 (61.0)	918 (53.2)		46.8 (44.4–49.1)	
Age group			< 0.001		< 0.001
49 years or younger	414 (14.6)	144 (34.8)		65.2 (60.4–69.6)	
50 to 59 years	684 (24.2)	311 (45.4)		54.6 (50.8–58.2)	
60 to 69 years	766 (27.1)	395 (51.6)		48.4 (44.9–51.9)	
70 years or older	962 (34.0)	600 (62.4)		37.6 (34.6–40.7)	
Schooling			< 0.001		< 0 .001
9 years or more	1,311 (46.3)	640 (48.8)		51.2 (48.4–53.9)	
8 years or less	1,146 (40.5)	665 (58.0)		42.0 (39.1–44.8)	
No information	372 (13.2)	145 (39.0)		61.0 (55.9–65.8)	
Race			< 0.001		< 0.001
White	2,472 (87.4)	1,282 (51.9)		48.1 (46.2–50.1)	
Others	172 (6.1)	108 (62.8)		37.2 (30.0–44.4)	
No information	185 (6.5)	60 (32.4)		67.6 (60.3–73.8)	
Marital status			< 0.001		< 0.001
With partner	1,598 (56.5)	824 (51.6)		48.4 (46.0–50.9)	
No partner	884 (31.3)	499 (56.5)		43.6 (40.3–46.8)	
No information	347 (12.3)	127 (36.6)		63.4 (58.1–68.2)	
Extension of disease			< 0.001		< 0.001
In situ	48 (1.7)	10 (20.8)		79.2 (64.7–88.2)	
Localized	541 (19.1)	173 (32.0)		68.0 (63.9–71.8)	
Metastatic	1,104 (39.0)	761 (68.9)		31.1 (28.4–33.8)	
Not applicable	28 (1.0)	17 (60.7)		39.3 (21.7–56.5)	
No information	1,108 (39.2)	489 (44.1)		55.9 (52.9–58.7)	
Grouping			< 0.001		< 0.001
Lip and oral cavity (C00-C08)	165 (5.8)	65 (39.4)		60.6 (52.7–67.6)	
Other pharynxes (C09-10; C12-14)	72 (2.6)	43 (59.7)		40.3 (29.0–51.3)	
Nasopharynx (C11)	24 (0.9)	12 (50.0)		50.0 (29.1–67.8)	
Esophagus (C15)	104 (3.7)	71 (67.6)		32.4 (23.7–41.4)	
Stomach (C16)	333 (11.8)	200 (60.1)		39.9 (34.7–45.2)	
Colon and rectum (C18-21)	813 (28.7)	291 (35.8)		64.2 (60.8–67.4)	
Liver (C22)	103 (3.6)	83 (80.6)		19.4 (12.5–27.6)	
Pancreas (C25)	112 (4.0)	96 (85.7)		14.3 (8.6–21.4)	
Larynx (C32)	88 (3.1)	36 (40.9)		59.1 (48.1–68.5)	
Trachea. Bronchi and Lungs (C33-C34)	527 (18.6)	407 (77.2)		22.8 (19.3–26.4)	
Kidney (C64-C66)	177 (6.3)	43 (24.3)		75.7 (68.7–81.4)	
Bladder (C67)	282 (10.0)	86 (30.5)		69.5 (63.8–74.5)	
Acute myeloid leukemia (C92.0)	28 (1.0)	17 (60.7)		39.3 (21.7–56.5)	
Year of diagnosis			0.623		0.690
2008	514 (18.2)	257 (50.0)		50.0 (45.6–54.2)	
2009	575 (20.3)	298 (51.8)		48.2 (44.0–52.2)	
2010	597 (21.1)	316 (52.9)		47.1 (43.0–51.0)	
2011	542 (19.2)	285 (52.5)		47.5 (43.3–51.6)	
2012	600 (21.2)	294 (49.0)		51.0 (46.9–54.9)	
Status					
Censorship	1,379 (48.8)				
Death	1,450 (51.3)				

^a^ p-value of the chi-square.

^b^ p-value of the Log-rank test.

S(t): probability of survival as a function of time; 95%CI: 95% confidence interval.

The cases included were mostly male (61.0%), over 70 years of age (34.0%), with 9 years of schooling or more (46.3%), of white race/color (87, 4%), with a partner (56.5%) and with a metastatic diagnosis (39.0%). During follow-up, 51.3% of cases died within five years. Except for the variable year of diagnosis, the others (gender, age group, schooling, race, marital status, extension of disease) were associated with death (Table 1). The most frequent type was colon and rectum (28.7%), followed by trachea, bronchi and lung (18.6%) and stomach (11.8%) (Table 1).

### Lip and oral cavity (C00-C08)

**Figure 1:**
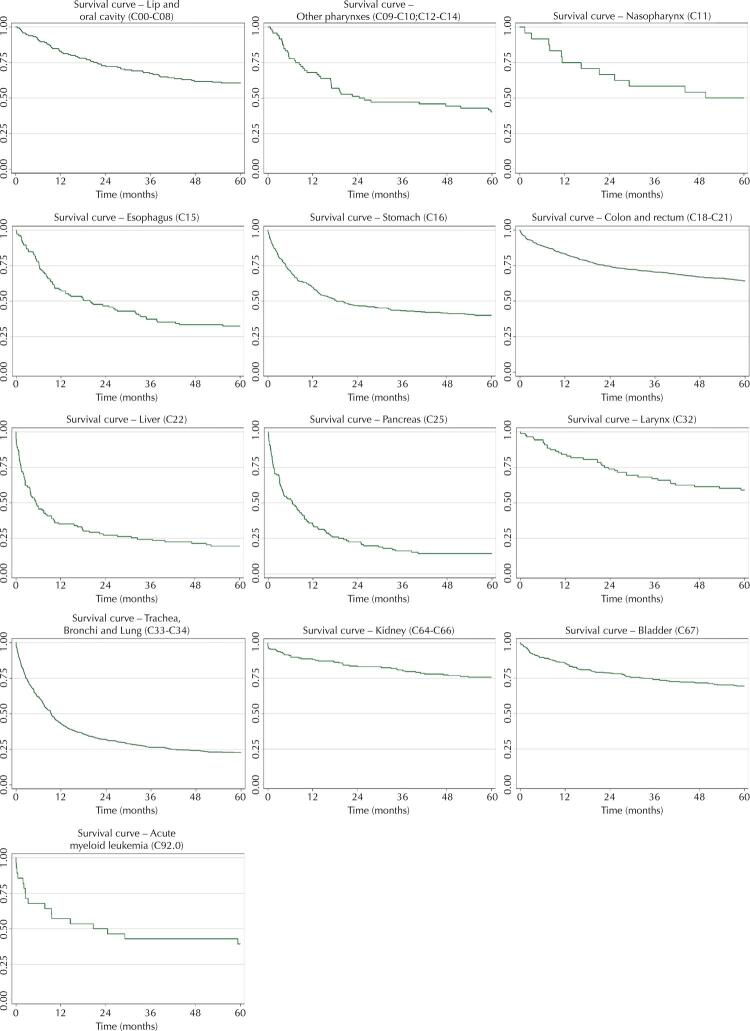
Survival of registered cases of tobacco-related cancer, Florianópolis, 2008–2017.

One hundred sixty-five (165) people with lip and oral cavity cancer were registered, or 5.8% of the total cases included. At the end of follow-up, 39.4% of them died and the probability of survival at the end was 60.6% (95%CI: 52.7–67.6) (Table 1 and Figure 1). It was more frequent in males, in people aged 50 to 59 years, with eight years of schooling or less, of the white race. Age range, race and disease extension were associated with survival. The adjusted analysis included the variables age, education, race, marital status and disease extension. Only other colors/races remained as a prognostic factor, with an increase of 2.6 in the risk of death (Table 2).

**Table 2:** Description, survival and proportional risks of death of registered cases of cancers of the lip and oral cavity (C00-C08), other pharynxes (C09-10; C12-14), nasopharynx (C11), larynx (C32), and trachea, bronchi and lung (C33-C34). Florianópolis, 2008–2017.

Variables	n (%)	Deaths	p^a^	S(t) (95%CI)	p^b^	HR (95%CI)	Adjusted HR (95%CI)

		n (%)					
**Lip and oral cavity (C00-C08)**^**c**^							
Sex			0.342		0.258		
Female	58 (35.2)	20 (34.5)		65.5 (51.8–76.2)		1.00	-
Male	107 (64.9)	45 (42.1)		57.9 (48.0–66.6)		1.35 (0.80–2.29)	-
Age group			0.016		0.022		
49 years or younger	35 (21.2)	10 (28.6)		71.4 (53.4–83.5)		1.00	1.00
50 to 59 years	48 (29.1)	22 (45.8)		54.2 (39.2–67.0)		1.77 (0.84–3.75)	1.40 (0.64–3.03)
60 to 69 years	35 (21.2)	8 (22.9)		77.1 (59.5–87.9)		0.75 (0.30–1.91)	0.63 (0.24–1.62)
70 years or older	47 (28.5)	25 (53.2)		46.8 (32.2–32.2)		2.14 (1.03–4.45)	1.94 (0.92–4.10)
Schooling			0.228		0.131		
9 years or more	69 (41.8)	22 (31.9)		68.1 (55.7–77.7)		1.00	1.00
8 years or less	84 (50.9)	37 (44.1)		56.0 (44.7–65.8)		1.50 (0.88–2.54)	1.13 (0.66–1.95)
No information	12 (7.3)	6 (50.0)		50.0 (20.9–73.6)		2.25 (0.91–5.56)	2.12 (0.48–9.27)
Race			0.040		0.015		
White	148 (89.7)	54 (36.5)		63.5 (55.2–70.7)		1.00	1.00
Others	9 (5.5)	7 (77.8)		22.2 (3.4–51.3)		2.80 (1.27–6.17)	2.61 (1.16–5.86)
No information	8 (4.9)	4 (50.0)		50.0 (15.2–77.5)		2.00 (0.72–5.52)	1.14 (0.20–6.45)
Marital status			0.929		0.948		
With partner	94 (57.0)	36 (38.3)		61.7 (51.1–70.7)		1.00	-
No partner	58 (35.2)	24 (41.4)		58.6 (44.9–70.0)		1.05 (0.63–1.76)	-
No information	13 (7.9)	5 (38.5)		61.5 (30.8–81.8)		1.15 (0.45–2.94)	-
Extension of disease			0.014		0.014		
Localized	34 (20.6)	12 (35.3)		64.7 (46.3–78.2)		1.00	1.00
Metastatic	57 (34.6)	31 (54.4)		45.6 (32.4–57.9)		1.75 (0.90–3.41)	1.76 (0.90–3.46)
No information	74 (44.9)	22 (29.7)		70.3 (58.5–79.3)		0.81 (0.40–1.64)	0.81 (0.39–1.66)
Year of diagnosis			0.842		0.851		
2008	28 (17.0)	13 (46.4)		53.6 (33.8–69.8)		1.00	-
2009	36 (21.8)	14 (38.9)		61.1 (43.4–74.8)		0.78 (0.36–1.65)	-
2010	32 (19.4)	12 (37.5)		62.5 (43.5–76.7)		0.75 (0.34–1.63)	-
2011	31 (18.8)	10 (32.3)		67.7 (48.4–81.2)		0.62 (0.27–0.42)	-
2012	38 (23.0)	16 (42.1)		57.9 (40.8–71.7)		0.79 (0.38–1.65)	-
**Cancer in other pharynxes (C09-C10; C12-C14)**							
Sex			0.717		0.936		
Female	6 (8.3)	4 (66.7)		33.3 (4.6–67.6)		1.00	-
Male	66 (91.7)	39 (59.1)		40.9 (29.0–52.4)		0.96 (0.34–2.68)	-
Age group				0.524		0.658	
49 years or younger	9 (12.5)	5 (55.6)		44.4 (13.6–71.9)		1.00	-
50 to 59 years	24 (33.3)	12 (50.0)		50.0 (29.1–67.8)		0.79 (0.28–2.24)	-
60 to 69 years	24 (33.3)	15 (62.5)		37.5 (19.0–56.0)		1.03 (0.38–2.85)	-
70 years or older	15 (20.8)	11 (73.3)		26.7 (8.3–49.6)		1.34 (0.46–3.85)	-
Schooling			0.733		0.816		
9 years or more	26 (36.1)	17 (65.4)		34.6 (17.5–52.5)		1.00	-
8 years or less	42 (58.3)	24 (57.1)		42.9 (27.8–57.1)		0.82 (0.44–1.52)	-
No information	4 (5.6)	2 (50.0)		50.0 (5.8–84.5)		0.85 (0.20–3.68)	-
Race			0.629		0.355		
White	62 (86.1)	36 (58.1)		41.9 (29.6–53.8)		1.00	-
Others	8 (11.1)	6 (75.0)		25.0 (3.7–55.8)		1.87 (0.78–4.45)	-
No information	2 (2.8)	1 (50.0)		50.0 (0.6–91.0)		1.01 (0.14–7.34)	-
Marital status			0.450		0.193		
With partner	46 (63.9)	25 (54.4)		45.7 (31.0–59.2)		1.00	-
No partner	22 (30.6)	15 (68.2)		31.8 (14.2–51.1)		1.49 (0.78–2.82)	-
No information	4 (5.6)	3 (75.0)		25.0 (0.9–66.5)		2.55 (0.76–8.50)	-
Extension of disease			0.573		0.492		
Localized	11 (15.3)	5 (45.5)		54.6 (22.9–78.0)		1.00	-
Metastatic	43 (59.7)	27 (62.8)		37.2 (23.1–51.3)		1.77 (0.68–4.59)	-
No information	18 (25.0)	11 (61.1)		38.9 (17.5–60.0)		1.70 (0.59–4.89)	-
Year of diagnosis			0.324		0.628		
2008	14 (19.4)	11 (78.6)		21.4 (5.2–44.8)		1.00	-
2009	21 (29.2)	12 (57.1)		42.9 (21.9–62.3)		0.67 (0.30–1.53)	-
2010	11 (15.3)	7 (63.6)		36.4 (11.2–62.7)		0.76 (0.29–1.95)	-
2011	13 (18.1)	5 (38.5)		61.5 (30.8–81.8)		0.36 (0.12–1.04)	-
2012	13 (18.1)	8 (61.5)		38.5 (14.1–62.8)		0.67 (0.27–1.67)	-
**Nasopharyngeal cancer (C11)**							
Sex			0.386		0.499		
Female	8 (33.3)	3 (37.5)		62.5 (22.9–86.1)		1.00	-
Male	16 (66.7)	9 (56.3)		43.8 (19.8–65.6)		1.56 (0.42–5.91)	-
Age group			0.050		0.062		
65 years or less	16 (66.7)	8 (50.0)		100.0		1.00	-
66 years or older	8 (33.3)	8 (50.0)		25.0 (0.9–66.5)		1.29 (0.39–4.29)	-
Schooling			1.000		0.996		
9 years or more	12 (50.0)	6 (50.0)		50.0 (20.9–73.6)		1.00	-
8 years or less	10 (41.7)	5 (50.0)		50.0 (18.4–75.3)		1.06 (0.32–3.46)	-
No information	2 (8.3)	1 (50.0)		50.0 (0.6–91.0)		1.01 (0.12–8.45)	-
Race			0.307		0.398		
White	23 (95.8)	12 (52.2)		47.8 (26.8–66.1)		-	
Others	1 (4.2)	-		-		-	
Marital status			0.231		0.312		
With partner	12 (50.0)	4 (33.3)		66.7 (33.7–86.0)		1.00	-
No partner	10 (41.7)	7 (70.0)		30.0 (7.1–57.8)		2.52 (0.74–8.64)	-
No information	2 (8.3)	1 (50.0)		50.0 (0.6–91.0)		1.80 (0.18–14.42)	-
Extension of disease			0.865		0.869		
Localized	5 (20.8)	2 (40.0)		60.0 (12.6–88.2)		1.00	-
Metastatic	11 (45.8)	6 (54.6)		45.5 (16.7–70.7)		1.54 (0.31–7.66)	-
No information	8 (33.4)	4 (50.0)		50.0 (15.2–77.5)		1.34 (0.25–7.35)	-
Year of diagnosis			0.525		0.601		
2008	6 (25.0)	4 (66.7)		33.3 (4.6–67.6)		1.00	-
2009	5 (20.8)	3 (60.0)		40.0 (5.2–75.3)		0.85 (0.19–3.80)	-
2010	8 (33.3)	4 (50.0)		50.0 (15.2–77.5)		0.54 (0.14–2.18)	-
2011	2 (8.3)	-		100.0		-	-
2012	3 (12.5)	1 (33.3)		66.7 (5.4–94.5)		0.44 (0.05–3.99)	-
**Laryngeal cancer (C32)**							
Sex			0.385		0.352		
Female	16 (18.2)	5 (31.3)		68.8 (40.5–85.6)		1.00	-
Male	72 (81.8)	31 (43.1)		56.9 (44.7–67.4)		1.56 (0.61–4.01)	-
Age group			0.430		0.434		
49 years or younger	11 (12.5)	2 (18.2)		81.8 (44.7–95.1)		1.00	-
50 to 59 years	33 (37.5)	14 (42.4)		57.6 (39.1–72.3)		2.94 (0.67–12.95)	-
60 to 69 years	22 (25.0)	10 (45.5)		54.6 (32.1–72.4)		3.23 (0.71–14.73)	-
70 years or older	22 (25.0)	10 (45.5)		54.6 (32.1–72.4)		3.18 (0.70–14.54)	-
Schooling			0.529		0.599		
9 years or more	43 (48.9)	15 (34.9)		65.1 (49.0–77.3)		1.00	-
8 years or less	34 (38.6)	16 (47.1)		52.9 (35.1–68.0)		1.47 (0.73–2.98)	-
No information	11 (12.5)	5 (45.5)		54.6 (22.9–78.0)		-	-
Race			0.529		0.974		
White	78 (88.6)	32 (41.0)		59.0 (47.3–68.9)		1.00	-
Others	7 (8.0)	3 (42.9)		57.1 (17.2–83.7)		1.44 (0.44–4.73)	-
No information	3 (3.4)	1 (33.3)		66.7 (5.4–94.5)		-	-
Marital status			0.924		0.924		
With partner	54 (61.4)	22 (40.7)		59.3 (45.0–71.0)		1.00	-
No partner	23 (26.1)	10 (43.5)		56.5 (34.3–73.8)		1.04 (0.49–2.22)	-
No information	11 (12.5)	4 (36.4)		63.6 (29.7–84.5)		-	-
Extension of disease			0.005		0.004		
Localized	31 (35.2)	7 (22.6)		77.4 (58.4–88.5)		1.00	-
Metastatic	28 (31.8)	18 (64.3)		35.7 (18.9–53.0)		3.79 (1.58–9.08)	-
No information	29 (33.0)	11 (40.9)		62.1 (42.1–76.9)		1.77 (0.69–4.57)	-
Year of diagnosis			0.337		0.383		
2008	15 (17.1)	8 (53.3)		46.7 (21.2–68.8)		1.00	-
2009	16 (18.2)	5 (31.3)		68.8 (40.5–85.6)		0.50 (0.16–1.54)	-
2010	22 (25.0)	11 (50.0)		50.0 (28.2–68.4)		0.83 (0.33–2.06)	-
2011	18 (20.5)	8 (44.4)		55.6 (30.5–74.8)		0.65 (0.24–1.72)	-
2012	17 (19.3)	4 (23.5)		76.5 (48.8–90.5)		0.34 (0.10–1.14)	-
**Cancer of the Trachea, Bronchi and Lungs (C33-C34)**^**d**^							
Sex			0.038		0.044		
Female	199 (37.8)	144 (72.4)		27.6 (21.6–34.0)		1.00	1.00
Male	328 (62.2)	263 (80.2)		19.8 (15.7–24.3)		1.23 (1.01–1.51)	1.19 (0.97–1.46)
age group			0.080		0.018		
49 years or younger	42 (8.0)	31 (73.8)		26.2 (14.1–40.0)		1.00	1.00
50 to 59 years	117 (22.2)	81 (69.2)		30.8 (22.7–39.2)		0.96 (0.63–1.45)	0.96 (0.63–1.46)
60 to 69 years	166 (31.6)	132 (79.5)		20.5 (14.7–26.9)		1.27 (0.86–1.88)	1.18 (0.80–1.75)
70 years or older	201 (38.2)	163 (81.1)		18.9 (13.8–24.6)		1.42 (0.97–2.09)	1.34 (0.92–1.98)
Schooling			0.258		0.013		
9 years or more	241 (45.7)	179 (74.3)		25.7 (20.4–31.4)		1.00	1.00
8 years or less	227 (43.1)	183 (80.6)		19.4 (14.5–24.8)		1.36 (1.11–1.67)	1.29 (1.05–1.59)
No information	59 (11.2)	45 (77.2)		23.7 (13.9–35.1)		1.10 (0.79–1.52)	1.29 (0.93–1.81)
Race			0.617		0.445		
White	478 (90.7)	368 (77.0)		23.0 (19.4–26.9)		1.00	-
Others	26 (4.9)	22 (84.6)		15.4 (4.8–31.5)		1.29 (0.84–1.98)	-
No information	23 (4.4)	17 (73.9)		26.1 (10.6–44.7)		0.89 (0.55–1.45)	-
Marital status			0.637		0.692		
With partner	309 (58.6)	243 (78.6)		21.4 (17.0–26.1)		1.00	-
No partner	168 (31.9)	127 (75.6)		24.4 (18.2–31.1)		0.99 (0.80–1.23)	-
No information	50 (9.5)	37 (74.0)		26.0 (14.9–38.6)		0.86 (0.61–1.22)	-
Extension of disease			< 0.001		< 0.001		
Localized	60 (11.4)	36 (60.0)		40.0 (27.7–52.0)		1.00	1.00
Metastatic	302 (57.3)	254 (84.1)		15.9 (12.0–20.3)		2.06 (1.45–2.92)	2.14 (1.40–2.85)
No information	165 (31.3)	117 (70.9)		29.1 (22.4–36.1)		1.34 (0.92–1.94)	1.38 (0.90–1.91)
Year of diagnosis			0.487		0.503		
2008	93 (17.7)	72 (77.4)		22.6 (14.7–31.5)		1.00	-
2009	112 (21.3)	92 (82.1)		17.9 (11.4–25.5)		1.17 (0.86–1.59)	-
2010	105 (19.9)	77 (73.3)		26.7 (18.6–35.4)		0.93 (0.68–1.29)	-
2011	102 (19.4)	81 (79.4)		20.6 (13.4–28.9)		1.18 (0.86–1.62)	-
2012	115 (21.8)	85 (73.9)		26.1 (18.5–34.3)		1.03 (0.76–1.42)	-

S(t): probability of survival as a function of time; 95%CI: 95% confidence interval; HR: proportional hazards ratio.

^a^ p-value of the c^2^ test (chi-square).

^b^ p-value of the Log-rank test..

^c^ HR adjusted for age group, race, education and disease extension.

^d^ HR adjusted for sex, age group, education and disease extension.

### Other pharynxes (C09-10; C12-14)

There were 72 cases related to other pharynxes, or 2.6% of the total. Of these, 59.7% died by the end of follow-up and the probability of survival was 40.3% (95%CI: 29.0–51.3) (Table 1 and Figure 1). It was more frequent in males, with less education and in those with a partner. No variable was associated with survival and no adjusted analysis was performed (Table 2).

### Nasopharynx (C11)

Nasopharyngeal diagnoses accounted for 0.9%, with 24 cases. Of these, 50% died and the probability of survival was 50.0% (95%CI: 29.1–67.8) (Table 1 and Figure 1). There were more records in men, aged 65 years or younger, and with nine years or more of schooling. Only the age range variable had a p-value < 0.20, in which the group aged 66 years or older showed a worse probability of survival (Table 2).

### Esophagus (C15)

In the period, there were 104 records of esophageal cancer, or 3.7% of the total, and 67.6% died. The probability of survival was 32.4% (95%CI: 23.7–41.1) (Table 1 and Figure 1). The worst survival was found among males, of other races/colors, without a partner, with a metastatic diagnosis. The adjusted analysis included the variables gender, race, marital status, disease extension, and year of diagnosis. Only other grouped colors/races remained as a worse prognostic factor, with an increase of 2.5 in the risk of death (Table 3).

**Table 3:** Description, survival and proportional risks of death of registered cases of esophageal (C15), stomach (C16), colon and rectum (C18-21), liver (C22) and pancreas (C25) cancers, Florianópolis, 2008–2017.

Variables	n (%)	Deaths	p^a^	S(t) (95%CI)	p^b^	HR (95%CI)	Adjusted HR (95%CI)

		n (%)					
**Esophageal cancer (C15)**^c^							
Sex			0.140		0.127		
Female	22 (21.0)	12 (54.6)		45.5 (24.4–64.3)		1.00	1.00
Male	83 (79.1)	59 (71.1)		28.9 (19.6–38.9)		1.62 (0.87–3.01)	1.64 (0.82–3.28)
Age group			0.577		0.351		
49 years or younger	9 (8.6)	7 (77.8)		22.2 (3.4–51.3)		1.00	-
50 to 59 years	39 (37.1)	28 (71.8)		28.2 (15.3–42.7)		0.64 (0.28–1.48)	-
60 to 69 years	24 (32.4)	20 (58.8)		41.2 (24.8–56.9)		0.47 (0.20–1.12)	-
70 years or older	23 (21.9)	16 (69.6)		30.4 (13.5–49.3)		0.67 (0.28–1.64)	-
Schooling			0.457		0.486		
9 years or more	31 (29.5)	21 (67.7)		32.3 (16.9–48.6)		1.00	-
8 years or less	56 (53.3)	40 (71.4)		28.6 (17.5–40.7)		1.34 (0.79–2.27)	-
No information	18 (17.1)	10 (55.6)		44.4 (21.6–65.1)		1.01 (0.47–2.14)	-
Race			0.015		0.002		
White	83 (79.1)	54 (65.1)		34.9 (24.9–45.1)		1.00	1.00
Others	16 (15.2)	15 (93.8)		6.3 (0.4–24.7)		2.54 (1.42–4.56)	2.50 (1.27–4.90)
No information	6 (5.7)	2 (33.3)		66.7 (19.5–90.4)		0.43 (0.10–1.76)	0.57 (0.09–3.35)
Marital status			0.073		0.074		
With partner	64 (61.0)	44 (68.8)		31.3 (20.4–42.7)		1.00	1.00
No partner	27 (25.7)	21 (77.8)		22.2 (9.0–39.0)		1.52 (0.90–2.56)	1.39 (0.80–2.43)
No information	14 (13.3)	6 (42.9)		57.1 (28.4–78.0)		0.57 (0.24–1.35)	1.26 (0.38–4.17)
Extension of disease			0.009		0.046		
Localized	24 (22.9)	13 (54.2)		45.8 (25.6–64.0)		1.00	1.00
Metastatic	37 (35.2)	32 (86.5)		13.5 (4.9–26.4)		1.96 (1.02–3.74)	2.09 (0.91–4.82)
No information	44 (41.9)	26 (59.1)		40.9 (26.5–54.8)		1.14 (0.59–2.22)	1.31 (0.58–2.96)
Year of diagnosis			0.012		0.021		
2008	20 (19.1)	9 (45.0)		55.0 (31.3–73.5)		1.00	1.00
2009	18 (17.1)	17 (94.4)		5.6 (0.4–22.4)		3.43 (1.51–7.82)	2.61 (0.96–7.05)
2010	18 (17.1)	10 (55.6)		44.4 (21.6–65.1)		1.32 (0.54–3.25)	1.25 (0.44–3.57)
2011	15 (14.3)	12 (80.0)		20.0 (4.9–42.4)		2.16 (0.91–5.15)	1.67 (0.56–4.94)
2012	34 (32.4)	23 (67.7)		32.4 (17.6–48)		1.72 (0.79–3.71)	1.23 (0.45–3.35)
**Stomach cancer (C16)**^**d**^							
Sex			0.099		0.094		
Female	142 (42.6)	78 (54.9)		45.1 (36.8–53.0)		1.00	1.00
Male	191 (57.4)	122 (63.9)		36.1 (29.4–42.9)		1.27 (0.96–1.69)	1.21 (0.91–1.61)
Age group			< 0.001		< 0.001		
59 years or younger	125 (37.5)	54 (43.2)		56.8 (47.7–64.9)		1.00	1.00
60 years or older	208 (62.5)	146 (70.2)		29.8 (23.7–36.1)		2.18 (1.59–2.98)	2.22 (1.62–3.03)
Schooling			0.214		0.229		
9 years or more	121 (36.3)	71 (58.7)		41.3 (32.5–49.9)		1.00	-
8 years or less	182 (54.7)	115 (63.2)		36.8 (29.9–43.8)		1.18 (0.88–1.59)	-
No information	30 (9.0)	14 (46.7)		53.3 (34.3–69.1)		0.78 (0.44–1.38)	-
Race			0.229		0.277		
White	293 (88.0)	177 (60.4)		39.6 (34.0–45.1)		1.00	-
Others	29 (8.7)	19 (65.5)		34.5 (18.2–51.5)		1.18 (0.74–1.90)	-
No information	11 (3.3)	4 (36.4)		63.6 (29.7–84.5)		0.50 (0.18–1.34)	-
Marital status			0.195		0.215		
With partner	209 (62.8)	124 (59.3)		40.7 (34.0–47.2)		1.00	-
No partner	98 (29.4)	64 (65.3)		34.7 (25.5–44.1)		1.16 (0.86–1.57)	-
No information	26 (7.8)	12 (46.2)		53.9 (33.3–70.6)		0.21 (0.38–1.24)	-
Extension of disease			< 0.001		0.002		
Localized	54 (16.2)	20 (37)		63.0 (48.7–74.3)		1.00	1.00
Metastatic	135 (40.5)	93 (68.9)		31.1 (23.5–39.0)		2.31 (1.42–3.75)	2.39 (1.46–3.88)
No information	144 (43.2)	87 (60.4)		39.6 (31.6–47.5)		2.00 (1.23–3.25)	1.92 (1.18–3.13)
Year of diagnosis			0.594		0.714		
2008	55 (16.5)	36 (65.5)		34.6 (22.4–47.0)		1.00	-
2009	54 (16.2)	34 (63.0)		37.0 (24.4–49.7)		0.95 (0.59–1.52)	-
2010	77 (23.1)	42 (54.6)		45.5 (34.1–56.1)		0.76 (0.49–1.19)	-
2011	75 (22.5)	42 (56.0)		44.0 (32.6–54.8)		0.80 (0.51–1.25)	-
2012	72 (21.6)	46 (63.9)		36.1 (25.2–47.1)		0.91 (0.59–1.40)	-
**Colon and rectal cancer (C18-21)**^**e**^							
Sex			0.742		0.906		
Female	403 (49.6)	142 (35.2)		64.8 (59.9–69.2)		1.00	-
Male	410 (50.4)	149 (36.3)		63.7 (58.8–68.1)		1.01 (0.81–1.28)	-
Age group			< 0.001		< 0.001		
49 years or younger	157 (19.3)	41 (26.1)		73.9 (66.3–80.0)		1.00	1.00
50 to 59 years	186 (22.9)	50 (26.9)		73.1 (66.1–78.9)		1.01 (0.67–1.53)	1.17 (0.77–1.78)
60 to 69 years	206 (25.3)	76 (36.9)		63.1 (56.1–69.3)		1.48 (1.02–2.17)	1.61 (1.10–2.37)
70 years or older	264 (32.5)	124 (47.0)		53.0 (46.8–58.8)		2.15 (1.51–3.05)	2.54 (1.77–3.65)
Schooling			< 0.001		< 0.001		
9 years or more	443 (54.5)	150 (33.9)		66.1 (61.5–70.3)		1.00	1.00
8 years or less	254 (31.2)	117 (46.1)		53.9 (47.6–59.8)		1.48 (1.17–1.89)	1.29 (1.01–1.66)
No information	116 (14.3)	24 (20.7)		79.3 (70.7–85.6)		0.58 (0.38–0.89)	0.64 (0.32–1.29)
Race			0.028		0.045		
White	703 (86.5)	262 (37.3)		62.7 (59.0–66.2)		1.00	1.00
Others	43 (5.3)	15 (34.9)		65.1 (49.0–77.3)		0.95 (0.56–1.60)	1.05 (0.62–1.80)
No information	67 (8.2)	14 (20.9)		79.1 (67.3–87.1)		0.51 (0.30–0.88)	1.32 (0.60–2.91)
Marital status			< 0.001		< 0.001		
With partner	436 (53.6)	150 (34.4)		65.6 (60.9–69.9)		1.00	1.00
No partner	268 (33.0)	117 (43.7)		56.3 (50.2–62.0)		1.41 (1.11–1.80)	1.33 (1.05–1.71)
No information	109 (13.4)	24 (22.0)		78.0 (69.0–84.7)		0.62 (0.40–0.95)	0.80 (0.38–1.66)
Extension of disease			< 0.001		< 0.001		
In situ	31 (3.8)	5 (16.1)		83.9 (65.5–93.0)		1.00	1.00
Localized	175 (21.5)	39 (22.3)		77.7 (70.8–83.2)		1.42 (0.56–3.61)	1.23 (0.49–3.16)
Metastatic	297 (36.5)	158 (53.2)		46.8 (41.0–52.4)		4.13 (1.69–10.06)	4.74 (1.92–11.66)
No information	310 (38.1)	89 (28.7)		71.3 (65.9–76.0)		1.94 (0.79–4.78)	1.95 (0.78–4.83)
Year of diagnosis			0.008		0.005		
2008	136 (16.7)	48 (35.3)		64.7 (56.1–72.1)		1.00	1.00
2009	176 (21.7)	55 (31.3)		68.8 (61.3–75.0)		0.85 (0.58–1.25)	0.50 (0.33–0.75)
2010	165 (20.3)	73 (44.2)		55.8 (47.9–63.0)		1.37 (0.95–1.97)	0.79 (0.53–1.18)
2011	148 (18.2)	62 (41.9)		58.1 (49.7–65.6)		1.20 (0.82–1.75)	0.55 (0.36–0.84)
2012	188 (23.1)	53 (28.2)		71.8 (64.8–77.7)		0.75 (0.51–1.11)	0.38 (0.25–0.59)
**Liver cancer (C22)**^**f**^							
Sex			0.727		0.407		
Female	29 (28.2)	24 (82.8)		17.2 (6.3–32.7)		1.00	-
Male	74 (71.8)	59 (79.7)		20.3 (12.0–30.0)		0.82 (0.51–1.32)	-
Age group			0.022		0.182		
49 years or younger	18 (17.5)	10 (55.6)		44.4 (21.6–65.1)		1.00	1.00
50 to 59 years	25 (24.3)	23 (92.0)		8.0 (1.4–22.5)		2.04 (0.97–4.31)	1.84 (0.86–3.94)
60 to 69 years	23 (22.3)	19 (82.6)		17.4 (5.4–35.0)		1.79 (0.83–3.86)	1.77 (0.82–3.82)
70 years or older	37 (35.9)	31 (83.8)		16.2 (6.6–29.6)		2.14 (1.05–4.38)	2.19 (1.06–4.53)
Schooling			0.579		0.678		
9 years or more	53 (51.4)	41 (77.4)		22.6 (12.5–34.6)		1.00	-
8 years or less	36 (35.0)	31 (86.1)		13.9 (5.1–27.1)		1.23 (0.77–1.96)	-
No information	14 (13.6)	11 (78.6)		21.4 (5.2–44.8)		1.07 (0.55–2.09)	-
Race			0.606		0.010		
White	94 (91.3)	75 (79.8)		20.2 (12.8–28.8)		1.00	1.00
Others	4 (3.8)	4 (100.0)		-		4.33 (1.53–12.26)	4.11 (1.40–12.07)
No information	5 (4.9)	4 (80.0)		20 (0.8–58.2)		1.07 (0.39–2.94)	0.85 (0.30–2.44)
Marital status			0.529		0.507		
With partner	54 (52.4)	44 (81.5)		18.5 (9.5–29.8)		1.00	-
No partner	36 (35.0)	30 (83.3)		16.7 (6.8–30.4)		1.15 (0.72–1.83)	-
No information	13 (12.6)	9 (69.2)		30.8 (9.5–55.4)		0.74 (0.36–1.52)	-
Extension of disease			0.284		0.746		
Localized	11 (10.7)	7 (63.6)		36.4 (11.2–62.7)		1.00	-
Metastatic	40 (38.8)	34 (85.0)		15.0 (6.1–27.6)		1.33 (0.59–3.00)	-
No information	52 (50.5)	42 (80.8)		19.2 (9.9–30.9)		1.36 (0.61–3.03)	-
Year of diagnosis			0.212		0.203		
2008	25 (24.2)	18 (72.0)		28.0 (12.4–46)		1.00	-
2009	19 (18.4)	14 (73.7)		26.3 (9.6–46.8)		1.01 (0.50–2.03)	-
2010	22 (21.4)	21 (95.5)		4.6 (0.3–18.9)		1.84 (0.98–3.47)	-
2011	15 (14.6)	11 (73.3)		26.7 (8.3–49.6)		1.05 (0.50–2.23)	-
2012	22 (21.4)	19 (86.4)		13.6 (3.4–30.9)		1.57 (0.82–3.00)	-
**Pancreatic cancer (C25)**^**f**^							
Sex			0.642		0.293		
Female	62 (55.4)	54 (87.1)		12.9 (6.0–22.5)		1.00	-
Male	50 (44.6)	42 (85.0)		16.0 (7.5–27.4)		0.81 (0.54–1.21)	-
Age group			0.340		0.072		
49 years or younger	10 (8.9)	7 (70.0)		30.0 (7.1–57.8)		1.00	1.00
50 to 59 years	24 (21.4)	20 (83.3)		16.7 (5.2–33.7)		1.30 (0.55–3.08)	1.19 (0.49–2.87)
60 to 69 years	24 (21.4)	20 (83.3)		16.7 (5.2–33.7)		1.21 (0.51–2.85)	1.19 (0.50–2.82)
70 years or older	54 (48.2)	49 (90.7)		9.3 (3.4–18.7)		2.05 (0.93–4.54)	1.91 (0.85–4.29)
Schooling			0.057		0.467		
9 years or more	50 (44.6)	46 (92.0)		8.0 (2.6–17.5)		1.00	-
8 years or less	54 (48.2)	42 (77.8)		22.2 (12.3–34)		0.79 (0.52–1.20)	-
No information	8 (7.2)	8 (100.0)		–		0.73 (0.34–1.54)	-
Race			0.236		0.638		
White	97 (86.6)	81 (83.5)		16.5 (9.9–24.5)		1.00	-
Others	10 (8.9)	10 (100.0)		–		1.35 (0.70–2.62)	-
No information	5 (4.5)	5 (100.0)		–		0.89 (0.36–2.21)	-
Marital status			0.987		0.623		
With partner	65 (58.0)	56 (86.2)		13.9 (6.8–23.3)		1.00	-
No partner	40 (35.7)	34 (85.0)		15.0 (6.1–27.6)		1.23 (0.80–1.88)	-
No information	7 (6.3)	6 (85.7)		14.3 (0.7–46.5)		0.97 (0.42–2.24)	-
Extension of disease			0.638		0.172		
Localized	8 (7.1)	7 (87.5)		12.5 (0.7–42.3)		1.00	1.00
Metastatic	73 (65.2)	64 (87.7)		12.3 (6.1–21.0)		1.75 (0.80–3.82)	1.48 (0.67–3.45)
No information	31 (27.7)	25 (80.7)		19.4 (7.9–34.6)		1.24 (0.54–2.87)	0.87 (0.45–2.59)
Year of diagnosis			0.764		0.885		
2008	14 (12.5)	12 (85.7)		14.3 (2.3–36.6)		1.00	-
2009	21 (18.8)	19 (90.5)		9.5 (1.6–26.1)		1.15 (0.56–2.37)	-
2010	28 (25.0)	25 (89.3)		10.7 (2.7–25.1)		1.09 (0.55–2.17)	-
2011	28 (25.0)	22 (78.6)		21.4 (8.7–37.8)		0.85 (0.42–1.72)	-
2012	21 (18.8)	18 (85.7)		14.3 (3.6–32.1)		1.06 (0.51–2.21)	-

S(t): probability of survival as a function of time; 95%CI: 95% confidence interval; HR: proportional hazards ratio.

^a^ p-value of the chi-square.

^b^ p-value of the Log-rank test.

^c^ HR adjusted for sex, race, marital status, disease extension, and year of diagnosis.

^d^ HR adjusted for sex, age group, and disease extent.

^e^ HR adjusted for age group, race, marital status, disease extension, and year of diagnosis.

^f^ HR adjusted for age group and disease extension.

### Stomach (C16)

Stomach cancer was the third most frequent, with 333 cases (11.8% of the total). Of these, 60.1% died, and the probability of survival was 39.9% (95%CI: 34.7–45.2) (Table 1 and Figure 1). The worst odds of survival were associated with being 60 years of age or older and having a metastatic diagnosis. The adjusted analysis included the variables gender, age group and disease extension. The risk of death was independently increased among those aged 60 years or older and those with a metastatic diagnosis or those recorded as having no information on disease extension (Table 3).

### Colon and rectum (C18-21)

Colon and rectal cancer was the most frequent, with 813 records (28.7% of the total), of which 35.8% died. The probability of survival at five years was 64.2% (95%CI: 60.8–67.4) (Table 1 and Figure 1). The worst survival rates were among people aged 70 years or older, with nine years or more of schooling, of white race/color, and with a metastatic diagnosis. In the adjusted analysis, being over 60 years of age, eight years or less of schooling, not having a partner and having a metastatic diagnosis independently increased the risk of death (Table 3).

### Liver (C22)

Liver cancer had 103 records (3.6% of the total), of which 80.6% died. This cancer was more frequent in men, white people and those with a partner. The 5-year survival probability was 19.4% (95%CI: 12.5–27.6), the second lowest (Table 1 and Figure 1). This was associated with age group and race. In the adjusted analysis, being 70 years of age or older increased the risk of death by 2.19, while being in the category other color/race increased it by four times (Table 3).

### Pancreas (C25)

Pancreatic cancer had 112 registered cases (4.0% of the total) and at follow-up, 85.7% died. It had the worst five-year survival probability, with 14.3% (95%CI: 8.6–21.4) (Table 1 and Figure 1). Survival probabilities were low in all categories of variables analyzed. Age group and disease extension were associated with survival. These variables were included in the adjusted analysis, but none independently increased the risk of death (Table 3).

### Larynx (C32)

Laryngeal cancer was recorded in 88 people (3.1% of the total). At follow-up, 40.9% died, and the 5-year survival probability was 59.1% (95%CI: 48.1–68.5) (Table 1 and Figure 1). There was a greater number of records in men, aged 50 to 59 years, with nine years or more of schooling and with a partner. No variables were associated with survival (Table 2).

### Trachea, bronchi and lungs (C33-C34)

Cancer of the trachea, bronchi and lung was the third most frequent, with 527 cases (18.6% of the total). At follow-up, 77.2% died and the 5-year survival probability was the third worst, 22.8% (95%CI: 19.3–26.4) (Table 1 and Figure 1). The diagnosis was more frequent in men, over 60 years of age, white, with a partner and metastatic extension. The adjusted analysis included the variables gender, age group, schooling and disease extension. The independent risk factors for death were eight years of schooling or less (18% increase) and metastatic staging (2.14 increase) (Table 2).

### Kidney (C64-C66)

Kidney cancer had 177 records (6.3% of the total). In five years, 24.3% of the sample died, and the probability of survival in the same period was 75.7% (95%CI: 68.7–81.4) (Table 1 and Figure 1). It was more frequent in men, of white race and with a partner. The worst survival rates were recorded among those aged 70 years or older, of other races/colors, without a partner and with a metastatic diagnosis. In the analysis adjusted for age, education, race, marital status and disease extension, the following were independent risk factors for death: being 70 years of age or older (HR = 3.77; 95%CI: 1.01–14.06) and being white (HR = 3.69; 95%CI: 1.20–11.29) (Table 4).

**Table 4:** Description, survival and proportional risks of death of registered cases of kidney (C64-C66), bladder (C67) and acute myeloid leukemia (C92.0) cancers, Florianópolis, 2008–2017.

Variables	n (%)	Deaths	p^a^	S(t) (95%CI)	p^b^	HR (95%CI)	Adjusted HR (95%CI)

		n (%)					
**Kidney cancer (C64-C66)**^**c**^							
Sex			0.421		0.441		
Female	71 (40.1)	15 (21.1)		78.9 (67.4–86.7)		1.00	-
Male	106 (59.9)	28 (26.4)		73.6 (64.1–80.9)		1.28 (0.68–2.39)	-
Age group			0.011		0.010		
49 years or younger	31 (17.5)	3 (9.7)		90.3 (72.9–96.8)		1.00	1.00
50 to 59 years	51 (28.8)	9 (17.7)		82.4 (68.8–90.4)		1.89 (0.51–6.99)	1.54 (0.40–5.96)
60 to 69 years	62 (35.0)	17 (27.4)		72.6 (59.7–82.0)		3.12 (0.92–10.66)	2.57 (0.73–9.12)
70 years or older	33 (18.6)	14 (42.4)		57.6 (39.1–72.3)		5.33 (1.53–18.55)	3.77 (1.01–14.06)
Schooling			0.129		0.158		
9 years or more	91 (51.4)	24 (26.4)		73.6 (63.3–81.5)		1.00	1.00
8 years or less	51 (28.8)	15 (29.4)		70.6 (56.0–81.1)		1.19 (0.62–2.26)	1.06 (0.54–2.07)
No information	35 (19.8)	4 (11.4)		88.6 (72.4–95.6)		0.42 (0.15–1.21)	0.94 (0.20–4.48)
Race			0.023		0.020		
White	152 (85.8)	38 (25.0)		75.0 (67.3–81.1)		1.00	1.00
Others	7 (4.0)	4 (57.1)		42.9 (9.8–73.4)		2.77 (0.99–7.78)	3.69 (1.20–11.29)
No information	18 (10.2)	1 (5.6)		94.4 (66.6–99.2)		0.20 (0.03–1.49)	0.40 (0.04–3.60)
Marital status			0.061		0.073		
With partner	92 (52.0)	23 (25.0)		75.0 (64.8–82.6)		1.00	1.00
No partner	44 (24.9)	15 (34.1)		65.9 (50.0–77.9)		1.45 (0.76–2.78)	1.57 (0.80–3.12)
No information	41 (23.8)	5 (12.2)		87.8 (73.2–94.7)		0.47 (0.18–1.23)	0.96 (0.24–3.87)
Extension of disease			< 0.001		< 0.001		
Localized	33 (18.6)	6 (18.2)		81.8 (63.9–91.4)		1.00	1.00
Metastatic	45 (25.4)	22 (48.9)		51.1 (35.8–64.5)		3.36 (1.36–8.29)	1.99 (0.75–5.29)
No information	99 (55.9)	15 (15.2)		84.9 (76.1–90.6)		0.81 (0.31–2.07)	0.57 (0.20–1.56)
Year of diagnosis			0.956		0.969		
2008	37 (20.9)	8 (21.6)		78.4 (61.4–88.6)		1.00	-
2009	34 (19.2)	9 (26.5)		73.5 (55.3–85.3)		1.31 (0.50–3.39)	-
2010	49 (27.7)	13 (26.5)		73.5 (58.7–83.6)		1.22 (0.51–2.95)	-
2011	34 (19.2)	7 (20.6)		79.4 (61.6–89.6)		0.99 (0.36–2.73)	-
2012	23 (13.0)	6 (26.1)		73.9 (50.9–87.3)		1.24 (0.43–3.56)	-
**Bladder cancer (C67)**^**d**^							
Sex			0.938		0.987		
Female	73 (25.9)	22 (30.1)		69.9 (57.9–79.0)		1.00	-
Male	209 (74.1)	64 (30.6)		69.4 (62.6–75.1)		1.00 (0.62–1.63)	-
Age group			0.001		< 0.002		
49 years or younger	25 (8.9)	2 (8.0)		92.0 (71.6–97.9)		1.00	1.00
50 to 59 years	55 (19.5)	12 (21.8)		78.2 (64.8–87.0)		2.85 (0.64–12.75)	3.00 (0.66–13.52)
60 to 69 years	71 (25.2)	17 (23.9)		76.1 (64.3–84.4)		3.21 (0.74–13.90)	3.25 (0.74–14.19)
270 years or older	131 (46.5)	55 (42.0)		58.0 (49.1–65.9)		6.22 (1.52–25.51)	6.19 (1.49–25.82)
Schooling			0.131		0.134		
9 years or more	115 (40.8)	35 (30.4)		69.6 (60.3–77.1)		1.00	1.00
8 years or less	109 (38.7)	39 (35.8)		64.2 (54.5–72.4)		1.24 (0.79–1.96)	1.09 (0.68–1.73)
No information	58 (20.6)	12 (20.7)		79.3 (66.5–87.7)		0.65 (0.34–1.26)	0.89 (0.36–2.19)
Race			0.129		0.164		
White	237 (84.0)	78 (32.9)		67.1 (60.7–72.7)		1.00	1.00
Others	12 (4.3)	2 (16.7)		83.3 (48.2–95.6)		0.47 (0.11–1.90)	0.65 (0.16–2.68)
No information	33 (11.7)	6 (18.2)		81.8 (63.9–91.4)		0.51 (0.22–1.18)	0.85 (0.27–2.68)
Marital status			0.368		0.256		
With partner	151 (53.6)	45 (29.8)		70.2 (62.2–76.8)		1.00	-
No partner	78 (27.7)	28 (35.9)		64.1 (52.4–73.6)		1.36 (0.85–2.18)	-
No information	53 (18.8)	13 (24.5)		75.5 (61.5–85.0)		0.83 (0.45–1.54)	-
Extension of disease			< 0.001		< 0.001		
In situ	17 (6.0)	5 (29.4)		70.6 (43.2–86.6)		1.00	1.00
Localized	95 (33.7)	19 (20.0)		80.0 (70.5–86.7)		0.66 (0.25–1.78)	0.59 (0.22–1.58)
Metastatic	36 (12.8)	22 (61.1)		38.9 (23.3–54.2)		2.79 (1.06–7.37)	2.38 (0.89–6.33)
No information	134 (47.5)	40 (29.9)		70.2 (61.6–77.1)		1.05 (0.41–2.66)	1.00 (0.39–2.56)
Year of diagnosis			0.567		0.605		
2008	67 (23.8)	16 (23.9)		76.1 (64.0–84.6)		1.00	-
2009	58 (20.6)	20 (34.5)		65.5 (51.8–76.2)		1.53 (0.79–2.96)	-
2010	54 (19.2)	18 (33.3)		66.7 (52.4–77.5)		1.44 (0.74–2.83)	-
2011	54 (19.2)	19 (35.2)		64.8 (50.6–75.9)		1.57 (0.81–3.05)	-
2012	49 (17.4)	13 (26.5)		73.5 (58.7–83.6)		1.13 (0.54–2.34)	-
**Acute myeloid leukemia (C92.0)**							
Sex			0.934		0.819		
Female	15 (53.6)	9 (60.0)		40.0 (16.5–62.8)		1.00	
Male	13 (46.4)	8 (61.5)		38.5 (14.1–62.8)		1.12 (0.43–2.90)	-
Age group			0.247		0.205		
65 years or less	15 (55.6)	8 (53.3)		46.7 (21.2–68.8)		1.00	
66 years or older	12 (44.4)	9 (75.0)		25.0 (6.0–50.5)		1.84 (0.71–4.81)	-
Schooling			0.010		0.015		
9 years or more	16 (57.1)	13 (81.3)		18.8 (4.6–40.3)		1.00	
8 years or less	7 (25.0)	1 (14.3)		85.7 (33.4–97.9)		0.09 (0.01–0.72)	-
No information	5 (17.9)	3 (60.0)		40.0 (5.2–75.3)		0.45 (0.12–1.61)	-
Race			0.444		0.432		
White	24 (85.7)	14 (62.5)		37.5 (19.0–56.0)		1.00	
Others	1 (3.6)	1 (100.0)		-		1.96 (0.25–15.37)	-
No information	3 (10.7)	1 (33.3)		66.7 (5.4–94.5)		0.35 (0.05–2.64)	-
Marital status			0.819		0.827		
With partner	12 (42.9)	8 (66.7)		33.3 (10.3–58.8)		1.00	
No partner	12 (42.9)	7 (58.3)		41.7 (15.3–66.5)		0.82 (0.30–2.27)	-
No information	4 (14.3)	2 (50.0)		50.0 (5.8–84.5)		0.63 (0.13–3.01)	-
Year of diagnosis			0.584		0.714		
2008	4 (14.3)	2 (50.0)		50.0 (5.8–84.5)		1.00	-
2009	5 (17.9)	4 (80.0)		20.0 (0.8–58.2)		1.62 (0.30–8.90)	-
2010	6 (21.4)	3 (50.0)		50.0 (11.1–80.4)		0.72 (0.12–4.34)	-
2011	8 (28.6)	6 (75.0)		25.0 (3.7–55.8)		1.40 (0.28–6.96)	-
2012	5 (17.9)	2 (40.0)		60.0 (12.6–88.2)		0.65 (0.09–4.62)	-

S(t): probability of survival as a function of time; 95%CI: 95% confidence interval; HR: proportional hazards ratio.

^a^ p-value of the chi-square.

^b^ p-value of the Log-rank test.

^c^ HR adjusted for age group, schooling, race, marital status and disease extension.

^d^ HR adjusted for age group, schooling, race and disease extension.

### Bladder (C67)

Bladder cancer had 282 recorded cases (10.0% of the total). In five years, 30.5% died and the probability of survival was 69.5% (95%CI: 63.8–74.5) (Table 1 and Figure 1). It was more frequent in men, aged 70 years or older, with nine years or more of schooling, white, with a partner and a localized diagnosis. The variables associated with survival were age group and disease extension. The adjusted analysis included the variables age, education, race and disease extension. Only being 70 years of age or older was an independent factor for a worse prognosis, with a six-fold increase in the risk of death (Table 4).

### Acute myeloid leukemia (C92.0)

AML was recorded in 28 cases and, of these, 60.7% died. At five years, the survival probability was 39.3% (95%CI: 21.7–56.5) (Table 1 and Figure 1). The distribution of cases was similar across sex and age, more frequent in those with nine years of schooling or more and who were white. None of the characteristics were associated with survival.

## DISCUSSION

The findings of the present research demonstrate that survival and prognostic factors vary according to each type of tobacco-related cancer in the city of Florianópolis (SC). The highest probability of survival was found in kidney cancer (75.7%), while the lowest in pancreatic (14.3%), liver (19.4%) and trachea, bronchi and lung cancer (22.8% %). The values obtained can be justified by the fact that longevity in Santa Catarina is 3.2 years above the national average, and the state has a triple burden of disease as an epidemiological profile of the elderly population, with a strong predominance of chronic conditions^13^. Brazil is among the countries with the greatest reduction in the prevalence of smoking in the population from 1990 to 2015, due to the adoption of public policies^3^. It should be noted that there is a higher prevalence of adult smokers aged 18 years and over (14.7%) in the South region, which contributes as a risk factor for the increase of these diseases, and many of the risk factors for death are smoking-related, such as being older and having less schooling.

The most frequent neoplasms found in this study were cancer of the colon and rectum, stomach and trachea, bronchi and lung, which is similar to the pattern found in the South and Southeast regions, placing them among the five most frequent types. Studies that analyze the survival and prognostic factors of these groups of neoplasms are scarce. Since they exhibit different behaviors, each type of cancer will be discussed separately.

### Lip and oral cavity cancer

The findings of this study show a survival probability of 57.9% and 65% for men and women, respectively. Another study carried out in Rio Grande do Norte, which only evaluated data on lip cancer, found that the 5-year survival rate was 88.9% for men and 87.3% for women^14^. People diagnosed with cancers of the lip, mouth and pharynx (ICD-10 C00-C14) had a survival rate of 33.3% at the end of 5-year follow-up in Santa Catarina, and staging at diagnosis was the greatest risk factor for death^15^. However, cases of lip and oral cavity were classified in the same group. In both studies, most cases were diagnosed in males and at an advanced age, corroborating these results. In the initial stage, cancer of the lip and oral cavity is usually asymptomatic, which leads to late diagnosis and metastasis^14,16^, worsening survival and quality of life.

### Oropharyngeal cancer

Research has shown a greater incidence of this type of cancer in males, aged between 50 and 60 years, and low survival rates, which is in line with the findings of this study^17,18^.

A study carried out in southern Brazil with the aim of observing survival after cancer of the oropharynx and mouth identified worse survival rates in individuals with oropharyngeal tumors, associating this data with the presence of cervical metastasis and greater tumor dissemination, in addition to location in regions of difficult visualization and diagnosis, which contributes negatively to survival rates^18^.

The literature also indicates that alcohol abuse and the presence of advanced stage disease act as main risk factors for worse survival outcomes^17^.

### Esophageal cancer

A systematic review carried out in 2015 showed that the 5-year survival for esophageal cancer varies between 10% and 15%, which is considered low. Also, that it is a rare disease before 30 years of age^19^. In the present study, the highest frequency was in the age group 50-59 years. A survey was also carried out with diagnostic data between 1997 and 2006 in Germany and the United States (USA), in which the 5-year survival was 18% in Germany and 17% in the USA^20^, lower than that found in this study. The low probability of survival from esophageal cancer is a reflection of late diagnosis, which in most cases is due to the absence of clinical signs in the initial stage^21^, although our results do not present worse prognostic factors.

### Stomach cancer

The 5-year survival rate for stomach cancer is around 31%, as found in studies carried out in the United States and Germany, which is consistent with the findings of this study^20,22^. In Florianópolis, survival rates below 50% were also found for both sexes (45.1% for women and 36.1% for men)^23^.

Despite advances in diagnosis and treatment, the disease remains highly lethal, the fifth most frequently diagnosed cancer and the third most lethal. Average survival rates reflect the fact that most people are diagnosed with the presence of metastases. It is a disease with histological and etiological heterogeneity^22^; therefore, primary prevention through the control of well-established risk factors (such as smoking, excessive alcohol consumption, high-salt food intake) is essential to reduce incidence rates, and warnings about signs and symptoms are essential for early diagnosis .

### Colon and rectal cancer

The findings of the present study are in line with research that identified an overall 5-year survival rate for people with colorectal cancer between 63 and 65% and worse prognosis in the age group over 70 years^23,24^.

Survival rates may vary depending on the stage and primary location of the disease. In metastatic or stage IV neoplasms, the relative survival rate is low, which was also identified in this study. Therefore, tumors in a metastatic setting make treatment more complex and tend to have a worse outlook^25^. In addition, it is noteworthy that the higher survival may be related to the early initiation of treatment, regardless of other characteristics^24^.

### Liver cancer

Despite some improvements in liver cancer survival rates in recent decades, the overall prognosis for this disease remains poor. A study carried out in the United States in 2017 reveals a 5-year survival rate of 21% in general, ranging from 4% for distant stage disease to 37% for localized disease^26^, which is close to the data found in this study.

The literature shows higher survival rates for these individuals, which may be related to better control of risk factors, early detection and treatment, pointing to the quality of health care provided^24^.

### Pancreatic cancer

Pancreatic cancer has a high lethality and despite not being among the ten most common types, it represents the eighth leading cause of death. In most cases, the diagnosis is made at a late stage, which makes the possibility of curative treatment difficult. The known survival rate is around 5% in five years^28^. In the present study, the survival rate was slightly higher, 12.9% for women and 16.0% for men, which still reflects a low survival rate.

As for independent risk factors for death, only advanced age was significant for these findings, which is supported by the literature in relation to the predominance of individuals affected in the sixth decade of life^28,29^.

### Bronchial, trachea and lung cancer

In this study, bronchial, trachea and lung cancer was the second most diagnosed. However, worldwide, this is the most incident type, with 2.1 million cases, affecting more men and usually detected in more advanced cases^4^.

Research carried out in Florianópolis showed similar results to those found here, with survival rates of 22.5%^23^. Such rates are higher than those reported in a survey carried out in Spain, which in five years reached 14%^30^.

The risk of lung cancer and consequent death from the disease is related to greater intensity of exposure to smoking, in addition to factors such as occupational exposure to chemical or physical agents^30^.

### Bladder cancer

Bladder cancer is the most frequent in the urinary tract and affects men more often, results similar to those of this study^4^. The 5-year overall survival identified in this study is between the 60% and 80% described in the literature ^23,31^.

Among newly diagnosed cases, approximately 30% have presented with locally advanced or metastatic disease. However, the population’s greater access to diagnostic imaging methods has increased early diagnoses, whose rates reflect much more favorable results^31^.

### Acute myeloid leukemia

AML is one of the four most frequent types of leukemia and the one that most affects adults^4^. The findings of this study are similar to those found in a study carried out in the United States, which showed that individuals diagnosed with AML and aged 75 years or older had lower survival rates compared to other age groups^32^.

Another study carried out in the USA shows that elderly people with AML have more comorbidities and higher rates of complications as compared to controls without cancer. Thus, the presence of comorbidities, previous organ dysfunction and a perception of unfitness for intensive chemotherapy interfere in the survival rates of this population^33^.

## FINAL CONSIDERATIONS

This study presented the survival rate and prognostic factors for 13 types of cancer that are tobacco-related. Worse prognostic factors and survival rates differ across types. Staging was the most frequent risk factor for worse prognosis, demonstrating the importance of campaigns to warn about primary prevention and early diagnosis. Smoking remains one of the main risk factors for several NCDs, including cancer, and thus directly impacts the burden of disease, with social and economic costs and loss of quality of life.

The study has limitations related to the use of secondary data, which results in the absence of information that could complement it, such as data on socioeconomic profile and smoking status. Likewise, the occurrence of incomplete data due to lack of information or typing error in the SIM makes data analysis difficult and can interfere with the research results. Also noteworthy is the lack of information about smoking during life, as this could reinforce for the population the importance of smoking as a risk factor for the development and evolution of the disease.

Among the positive points we highlight the RCBP quality indices, the percentage of histological verification – 92.6%, the percentage of cases identified only by the death certificate – 2.68%, and the percentage of cases diagnosed with unknown primary location (C80.9) – 0.83%, during the period from 2008 to 2012. In addition, the Santa Catarina SIM has high completeness.

Florianópolis follows the behavior of the south of the country, as already identified in other studies, such as the international panorama for some types of cancer. The National Policy for Cancer Prevention and Control is based on data from national and international studies to guide its actions. In this sense, this study can contribute to the adaptation of policies and actions to local realities, since there is a very large sociocultural, economic and population diversity in the country. Such realities directly impact the data found in the different Brazilian regions. For cancer control, it is essential to know the local characteristics, which dialogue with the social characteristics and access to health in the territory. Understanding the behavior of the disease through survival analysis is one of the ways to support the accomplishment of prevention, promotion and awareness work.

The findings of this study also reinforce the need for public investment in tobacco control policy, in order to expand and guarantee the population’s access to policies at the local level, since all the neoplasms discussed here are related to tobacco use and integrate NCD coping plans. Despite the laws that support cases of suspicion or diagnosis of neoplasia, the anti-smoking law of the municipality of Florianópolis and the Tobacco Control Policy, it is necessary to extrapolate the data found to health professionals and managers, aiming at strengthening and consolidating the measures of prevention, early detection, screening and appropriate treatment for cancer.
