# Supplementary material for: Tobacco-related neoplasms: survival analysis and risk of death of population data from Florianópolis, SC
Source: Rev Saude Publica. 2022 Mar 29;56:16. doi: 10.11606/s1518-8787.2022056003651 (PMC8973025; doi:10.11606/s1518-8787.2022056003651)
Supplement: Translation

# Translation: Neoplasias relacionadas ao tabaco: análise de sobrevivência e risco de óbito de dados populacionais de Florianópolis, SC

**Authors:** Ione Jayce Ceola Schneider, Tauana Prestes Schmidt, Vanessa Pereira Correa, Ana Maria Martins dos Santos, Bruna Vanti da Rocha, Leandro Pereira Garcia, Roger Flores Ceccon

**Affiliations:** I Brasil Universidade Federal de Santa Catarina. Departamento de Ciências da Saúde. Araranguá, SC, Brasil; II Brasil Universidade Federal de Santa Catarina. Programa de Pós-Graduação em Ciências da Reabilitação. Araranguá, SC, Brasil; III Brasil Universidade Federal de Santa Catarina. Programa de Pós-Graduação em Saúde Coletiva. Florianópolis, SC, Brasil; IV Brasil Secretaria Municipal de Saúde de Florianópolis. Florianópolis, SC, Brasil

**Keywords:** Tabagismo, Neoplasias, epidemiologia, Análise de Sobrevida, Fatores de Risco, Mortalidade, Modelos de Riscos Proporcionais

## Abstract

**OBJETIVO:**

Estimar a probabilidade de sobrevivência e os fatores prognósticos das neoplasias relacionados ao tabagismo em uma coorte de base populacional.

**MÉTODOS:**

Trata-se de uma coorte com dados do Registro de Câncer de Base Populacional de Florianópolis, região Sul do Brasil, de 2008 a 2012. Utilizou-se o *software Stata* 16.0 para estimar as probabilidades de sobrevivência em cinco anos após o diagnóstico, pelo método de Kaplan Meier, e os riscos de óbito, pela regressão de Cox.

**RESULTADOS:**

Foram incluídos 2.829 registros de câncer relacionados ao tabagismo, mais prevalentes entre pessoas do sexo masculino, com mais de 70 anos, nove anos ou mais de escolaridade, cor branca, com companheiro e diagnóstico metastático. Os agrupamentos mais frequentes foram cólon e reto (28,7%), traqueia, brônquios e pulmões (18,6%) e estômago (11,8%). No acompanhamento, 1.450 foram a óbito. O câncer de pâncreas foi o que apresentou pior probabilidade de sobrevivência (14,3%), seguido pelo câncer de fígado (19,4%).

**CONCLUSÃO:**

Os fatores de risco para o óbito e as taxas de sobrevivência diferem entre os 13 tipos de câncer relacionados ao tabaco. O diagnóstico precoce e a prevenção primária são estratégias que devem ser aprimoradas para melhorar a sobrevivência e diminuir a carga relacionada a esses tipos de câncer.

## INTRODUÇÃO

O tabagismo caracteriza-se como um importante problema de saúde pública e é considerado o segundo fator de risco modificável mais prevalente para doenças crônicas não transmissíveis (DCNT) e para a mortalidade global. No Brasil, atualmente, são mais de 7 milhões de fumantes ativos e 1,2 milhão de fumantes passivos. Além do mais, 428 pessoas morrem diariamente em decorrência da dependência da nicotina, 56,9 bilhões de reais são gastos anualmente com despesas médicas e perda de produtividade e 156.216 mortes anuais poderiam ser evitadas pela diminuição do uso do tabaco^1^.

A incidência de câncer relacionado ao tabaco tem aumentado de maneira significativa nos últimos anos, principalmente tumores na cavidade oral, faringe, esôfago, estômago, cólon, reto, fígado, pâncreas, laringe, pulmão, brônquios, traqueia, rins, bexiga urinária e colo uterino, além de leucemia mieloide aguda (LMA)^2^. Em 2015, o câncer foi uma das principais causas de anos de vida perdidos por incapacidade atribuíveis ao tabagismo e constituiu-se como a causa de 20% do total de mortes pela doença^3^.

No mundo, é definida que 18 milhões de pessoas foram diagnosticadas com câncer e 9,6 milhões foram a óbito no ano de 2018, a maioria do sexo masculino e em países de baixa e média renda^4^. No Brasil, a incidência é de mais 500 mil novos casos, a mortalidade ultrapassou 100 mil óbitos no mesmo período e a taxa de sobrevida esperada para todos os cânceres é de aproximadamente 50% em cinco anos^4,5^.

A sobrevida é definida como o período pelo qual um paciente permanece vivo após o diagnóstico da doença ou início do tratamento. Embora seja um importante indicador para mensurar a magnitude do tabagismo e do câncer na população, a sobrevida após o diagnóstico de tumores causados pelo tabaco ainda é desconhecida no Brasil. Além do mais, a análise da sobrevida possibilita avaliar as ações na área oncológica implementadas no país nos últimos anos, bem como seus fatores prognósticos. Ainda, o estudo da sobrevida permite conhecer o comportamento da doença, possibilitando uma abordagem que proporcione melhor qualidade de vida aos indivíduos acometidos^6^.

Há poucos relatos na literatura científica acerca da sobrevivência de pessoas com câncer relacionado ao tabaco, embora evidencie-se que o maior consumo de cigarro está associado à pior progressão da doença^2^.

Assim, o objetivo deste trabalho foi estimar a probabilidade de sobrevivência e os fatores prognósticos das neoplasias relacionadas ao tabagismo em uma coorte de base populacional.

## MÉTODOS

Trata-se de uma coorte de base populacional com dados do Registro Municipal de Câncer de Florianópolis, município localizado na região Sul do país. É a capital do estado de Santa Catarina, tem aproximadamente 500 mil habitantes e caracteriza-se por seu Índice de Desenvolvimento Humano (IDH) muito alto: 0,815, atribuído principalmente à longevidade e renda^7^. O município conta com alta cobertura e experiência consolidada na atenção primária, refletindo em bons resultados de rastreamento e detecção precoce das condições de saúde de sua população. Além disso, o atendimento aos pacientes oncológicos, na média e alta complexidade, é contemplado por mais de seis centros hospitalares, além de associações que se dedicam à conscientização e busca por direitos das pessoas com câncer.

A população do estudo é composta por indivíduos cadastrados no Registro de Câncer de Base Populacional de Florianópolis, no período de 1 de janeiro de 2008 a 31 de dezembro de 2012, com diagnóstico de neoplasia relacionada ao tabagismo. Os cânceres incluídos foram aqueles com código de Classificação Internacional de Doenças para a Oncologia (CID-O)^8^ considerado relacionado ao tabagismo^9^. Entre eles estão os de lábio e cavidade oral (C00-C08); nasofaringe (C11); outras faringes (C09-C10, C12-C14); esôfago (C15); estômago (C16); cólon e reto (C18-C21); fígado (C22); pâncreas (C25); laringe (C32); traqueia, brônquios e pulmões (C33-C34); rim (C64-C66); bexiga (C67) e leucemia mieloide aguda (C92.0). Foram excluídos os casos que tiveram o meio de diagnóstico registrado como não informado ou que o diagnóstico foi exclusivamente realizado pelo serviço de verificação de óbito (SVO). Os casos de câncer de colo do útero e ovário, apesar de serem relacionados ao tabaco, não foram incluídos nas análises, mesmo apresentando o tabaco como fator causal. O muco cervical de fumantes é mais mutagênico e sugere associação com danos no DNA em mulheres com câncer de colo do útero. Já no câncer de ovário, os dados sugerem estar associado apenas ao tipo mucinoso, no qual o tabagismo pode contribuir para uma progressão da forma benigna para a maligna mais rapidamente. Esses dois últimos não foram adicionados às análises por se referirem exclusivamente ao sexo feminino e o Brasil ter políticas públicas específicas para saúde da mulher, especialmente com ação estratégica de rastreamento do câncer de colo de útero.

Para possibilitar o acompanhamento dos casos registrados no RCBP e estimar a sobrevivência, são necessárias informações referentes aos óbitos, como data e causa básica. Para isso, foi utilizado o Sistema de Informação sobre Mortalidade (SIM) do Ministério da Saúde, com as informações referentes ao município de Florianópolis no período de 1 de janeiro de 2008 a 31 de dezembro de 2017. A criação do banco de dados foi fundamentada na técnica de relacionamento probabilístico de registros, que permite a integração de bases de dados de natureza diversa, contribuindo para a melhoria da qualidade dos dados registrados^12^. Para tal, utilizou-se o *software* OpenRecLink 3.1. Para o pareamento dos dados, foram utilizadas as seguintes variáveis do SIM: número da declaração de óbito, nome, data de nascimento, nome da mãe, data de óbito, causa básica da morte; e do banco do RCBP: número do registro, nome, data de nascimento, nome da mãe. Os parâmetros do relacionamento seguiram as instruções do *software*. Após o relacionamento dos bancos de dados, foi criado um banco único contendo todas as informações pertinentes ao estudo, permitindo a análise dos dados.

O tempo de sobrevivência foi calculado como o intervalo, em meses, entre a data do diagnóstico e a data do óbito ou final do acompanhamento. O tempo máximo de acompanhamento foi de 60 meses. Todos os casos não encontrados no SIM foram considerados vivos ao final do acompanhamento e censurados.

As variáveis independentes foram sexo (feminino, masculino), faixa etária (com pontos de corte para cada tipo de câncer), raça/cor (branca, preto/pardo/indígena/amarelo, sem informação), escolaridade (9 anos ou mais, 8 anos ou menos, sem informação), estado conjugal (com companheiro, sem companheiro, sem informação), extensão do câncer (localizado, metástase, sem informação) e ano do diagnóstico (2008, 2009, 2010, 2011, 2012).

Inicialmente, foi realizada análise descritiva com frequência absoluta e relativa das variáveis independentes. Após, foi realizada a análise bivariada entre as variáveis independentes e a variável status (censura/óbito) para estimar a incidência de óbitos em cada categoria e utilizado o teste qui-quadrado de Pearson para testar a associação entre as variáveis. Essas análises foram realizadas para todos os casos e para cada tipo de câncer.

Para cada tipo de câncer, a probabilidade de sobrevivência foi estimada pelo método de Kaplan-Meier. Para comparar as curvas de sobrevivência estratificadas pelas variáveis independentes, foi utilizado o teste log-rank. A estimação do efeito das variáveis independentes foi realizada pelo modelo semiparamétrico de riscos proporcionais de Cox bruta e ajustada. As variáveis com valor de p < 0,20 foram incluídas na análise ajustada. Para a análise dos dados e construção dos gráficos de sobrevida, foi utilizado o programa estatístico *Stata* (*Stata Corp, v.16.0. Stata Statistical Software. College Station: StataCorp LP*, 2021).

Esta pesquisa foi fundamentada nos princípios éticos, com base na Resolução n° 466, de 12 de dezembro de 2012, do Conselho Nacional de Saúde, e aprovada sob o CAAE: 53518116.1.0000.0121.

Esse trabalho é resultado do projeto financiado pela Fundação de Amparo à Pesquisa e Inovação do Estado de Santa Catarina, no Programa de Pesquisa para o SUS: Gestão Compartilhada em Saúde (PPSUS) (Termo de Outorga n° 2016RT2206).

## RESULTADOS

### Descrição Geral

No período de 1 de janeiro de 2008 a 31 de dezembro de 2012, 3.012 casos de câncer relacionados ao tabaco foram registrados pelo RCBP no município de Florianópolis. Desses, 189 foram excluídos por terem sido diagnosticados pelo SVO ou pelo método diagnóstico não estar disponível. Dessa forma, a população deste estudo é composta por 2.829 registros (Tabela 1).

**Tabela 1:** Descrição e sobrevivência dos casos registrados de câncer relacionado ao tabaco, Florianópolis, 2008–2017.

Variáveis	n (%)	Óbitos	p^a^	S(t) (IC95%)	p^b^

		n (%)			
Sexo			0,009		0,019
Feminino	1.104 (39,0)	532 (48,2)		51,8 (48,8–54,7)	
Masculino	1.725 (61,0)	918 (53,2)		46,8 (44,4–49,1)	
Faixa etária			< 0,001		< 0,001
49 anos ou menos	414 (14,6)	144 (34,8)		65,2 (60,4–69,6)	
50 a 59 anos	684 (24,2)	311 (45,4)		54,6 (50,8–58,2)	
60 a 69 anos	766 (27,1)	395 (51,6)		48,4 (44,9–51,9)	
70 anos ou mais	962 (34,0)	600 (62,4)		37,6 (34,6–40,7)	
Escolaridade			0,001		< 0 ,001
9 anos ou mais	1.311 (46,3)	640 (48,8)		51,2 (48,4–53,9)	
8 anos ou menos	1.146 (40,5)	665 (58,0)		42,0 (39,1–44,8)	
Sem informação	372 (13,2)	145 (39,0)		61,0 (55,9–65,8)	
Raça			< 0,001		< 0,001
Branca	2.472 (87,4)	1.282 (51,9)		48,1 (46,2–50,1)	
Outras	172 (6,1)	108 (62,8)		37,2 (30,0–44,4)	
Sem informação	185 (6,5)	60 (32,4)		67,6 (60,3–73,8)	
Situação conjugal			< 0,001		< 0,001
Com companheiro	1.598 (56,5)	824 (51,6)		48,4 (46,0–50,9)	
Sem companheiro	884 (31,3)	499 (56,5)		43,6 (40,3–46,8)	
Sem informação	347 (12,3)	127 (36,6)		63,4 (58,1–68,2)	
Extensão da doença			< 0,001		< 0,001
In situ	48 (1,7)	10 (20,8)		79,2 (64,7–88,2)	
Localizado	541 (19,1)	173 (32,0)		68,0 (63,9–71,8)	
Metastático	1.104 (39,0)	761 (68,9)		31,1 (28,4–33,8)	
Não se aplica	28 (1,0)	17 (60,7)		39,3 (21,7–56,5)	
Sem informação	1.108 (39,2)	489 (44,1)		55,9 (52,9–58,7)	
Agrupamento			< 0,001		< 0,001
Lábio e cavidade oral (C00-C08)	165 (5,8)	65 (39,4)		60,6 (52,7–67,6)	
Outras faringes (C09-10; C12-14)	72 (2,6)	43 (59,7)		40,3 (29,0–51,3)	
Nasofaringe (C11)	24 (0,9)	12 (50,0)		50,0 (29,1–67,8)	
Esôfago (C15)	104 (3,7)	71 (67,6)		32,4 (23,7–41,4)	
Estômago (C16)	333 (11,8)	200 (60,1)		39,9 (34,7–45,2)	
Colón e reto (C18-21)	813 (28,7)	291 (35,8)		64,2 (60,8–67,4)	
Fígado (C22)	103 (3,6)	83 (80,6)		19,4 (12,5–27,6)	
Pâncreas (C25)	112 (4,0)	96 (85,7)		14,3 (8,6–21,4)	
Laringe (C32)	88 (3,1)	36 (40,9)		59,1 (48,1–68,5)	
Traqueia, brônquios e pulmão (C33-C34)	527 (18,6)	407 (77,2)		22,8 (19,3–26,4)	
Rim (C64-C66)	177 (6,3)	43 (24,3)		75,7 (68,7–81,4)	
Bexiga (C67)	282 (10,0)	86 (30,5)		69,5 (63,8–74,5)	
Leucemia mieloide aguda (C92.0)	28 (1,0)	17 (60,7)		39,3 (21,7–56,5)	
Ano do diagnóstico			0,623		0,690
2008	514 (18,2)	257 (50,0)		50,0 (45,6–54,2)	
2009	575 (20,3)	298 (51,8)		48,2 (44,0–52,2)	
2010	597 (21,1)	316 (52,9)		47,1 (43,0–51,0)	
2011	542 (19,2)	285 (52,5)		47,5 (43,3–51,6)	
2012	600 (21,2)	294 (49,0)		51,0 (46,9–54,9)	
Status					
Censura	1.379 (48,8)				
Óbito	1.450 (51,3)				

^a^ Valor de p do teste qui-quadrado.

^b^ Valor de p do teste Log-rank.

S(t): probabilidade de sobrevivência em função do tempo; IC95%: intervalo de confiança de 95%.

Os casos incluídos foram em sua maioria do sexo masculino (61,0%), acima dos 70 anos (34,0%), com 9 anos ou mais de estudo (46,3%), da raça/cor branca (87,4%), com companheiro (56,5%) e com diagnóstico metastático (39,0%). No acompanhamento, 51,3% dos casos foram a óbito no período de cinco anos. Com exceção da variável ano do diagnóstico, as demais (sexo, faixa etária, escolaridade, raça, situação conjugal, extensão da doença) foram associadas ao óbito (Tabela 1). O tipo mais frequente foi de cólon e reto (28,7%), seguido pelo de traqueia, brônquios e pulmão (18,6%) e estômago (11,8%) (Tabela 1).

### Lábio e cavidade oral (C00-C08)

Foram registradas 165 pessoas com câncer de lábio e cavidade oral, ou 5,8% do total de casos incluídos. Ao final do acompanhamento, 39,4% delas foram a óbito e a probabilidade de sobrevivência ao final foi de 60,6% (IC95% 52,7–67,6) (Tabela 1 e Figura 1). Foi mais frequente no sexo masculino, em pessoas de 50 a 59 anos, com oito anos ou menos de escolaridade, da raça branca. Faixa etária, raça e extensão da doença foram associadas à sobrevivência. Na análise ajustada, foram incluídas as variáveis faixa etária, escolaridade, raça, situação conjugal e extensão da doença. Apenas outras cores/raças se mantiveram como fator prognóstico, com aumento de 2,6 no risco de óbito (Tabela 2).

**Figura 1:** Sobrevivência dos casos registrados de câncer relacionado ao tabaco, Florianópolis, 2008–2017.

**Tabela 2:** Descrição, sobrevivência e riscos proporcionais de óbito dos casos registrados de cânceres de lábio e cavidade oral (C00-C08), outras faringes (C09-10; C12-14), nasofaringe (C11), laringe (C32) e traqueia, brônquios e pulmão (C33-C34). Florianópolis, 2008–2017.

Variáveis	n (%)	Óbitos	p^a^	S(t) (IC95%)	p^b^	HR bruta (IC95%)	HR ajustada (IC95%)

		n (%)					
**Lábio e cavidade oral (C00-C08)**^**c**^							
Sexo			0,342		0,258		
Feminino	58 (35,2)	20 (34,5)		65,5 (51,8–76,2)		1,00	-
Masculino	107 (64,9)	45 (42,1)		57,9 (48,0–66,6)		1,35 (0,80–2,29)	-
Faixa etária			0,016		0,022		
49 anos ou menos	35 (21,2)	10 (28,6)		71,4 (53,4–83,5)		1,00	1,00
50 a 59 anos	48 (29,1)	22 (45,8)		54,2 (39,2–67,0)		1,77 (0,84–3,75)	1,40 (0,64–3,03)
60 a 69 anos	35 (21,2)	8 (22,9)		77,1 (59,5–87,9)		0,75 (0,30–1,91)	0,63 (0,24–1,62)
70 anos ou mais	47 (28,5)	25 (53,2)		46,8 (32,2–32,2)		2,14 (1,03–4,45)	1,94 (0,92–4,10)
Escolaridade			0,228		0,131		
9 anos ou mais	69 (41,8)	22 (31,9)		68,1 (55,7–77,7)		1,00	1,00
8 anos ou menos	84 (50,9)	37 (44,1)		56,0 (44,7–65,8)		1,50 (0,88–2,54)	1,13 (0,66–1,95)
Sem informação	12 (7,3)	6 (50,0)		50,0 (20,9–73,6)		2,25 (0,91–5,56)	2,12 (0,48–9,27)
Raça			0,040		0,015		
Branca	148 (89,7)	54 (36,5)		63,5 (55,2–70,7)		1,00	1,00
Outras	9 (5,5)	7 (77,8)		22,2 (3,4–51,3)		2,80 (1,27–6,17)	2,61 (1,16–5,86)
Sem informação	8 (4,9)	4 (50,0)		50,0 (15,2–77,5)		2,00 (0,72–5,52)	1,14 (0,20–6,45)
Situação conjugal			0,929		0,948		
Com companheiro	94 (57,0)	36 (38,3)		61,7 (51,1–70,7)		1,00	
Sem companheiro	58 (35,2)	24 (41,4)		58,6 (44,9–70,0)		1,05 (0,63–1,76)	
Sem informação	13 (7,9)	5 (38,5)		61,5 (30,8–81,8)		1,15 (0,45–2,94)	
Extensão da doença			0,014		0,014		
Localizado	34 (20,6)	12 (35,3)		64,7 (46,3–78,2)		1,00	1,00
Metastático	57 (34,6)	31 (54,4)		45,6 (32,4–57,9)		1,75 (0,90–3,41)	1,76 (0,90–3,46)
Sem informação	74 (44,9)	22 (29,7)		70,3 (58,5–79,3)		0,81 (0,40–1,64)	0,81 (0,39–1,66)
Ano do diagnóstico			0,842		0,851		
2008	28 (17,0)	13 (46,4)		53,6 (33,8–69,8)		1,00	
2009	36 (21,8)	14 (38,9)		61,1 (43,4–74,8)		0,78 (0,36–1,65)	
2010	32 (19,4)	12 (37,5)		62,5 (43,5–76,7)		0,75 (0,34–1,63)	
2011	31 (18,8)	10 (32,3)		67,7 (48,4–81,2)		0,62 (0,27–0,42)	
2012	38 (23,0)	16 (42,1)		57,9 (40,8–71,7)		0,79 (0,38–1,65)	
**Câncer em outras faringes (C09-C10; C12-C14)**							
Sexo			0,717		0,936		
Feminino	6 (8,3)	4 (66,7)		33,3 (4,6–67,6)		1,00	-
Masculino	66 (91,7)	39 (59,1)		40,9 (29,0–52,4)		0,96 (0,34–2,68)	-
Faixa etária			0,524		0,658		
49 anos ou menos	9 (12,5)	5 (55,6)		44,4 (13,6–71,9)		1,00	-
50 a 59 anos	24 (33,3)	12 (50,0)		50,0 (29,1–67,8)		0,79 (0,28–2,24)	-
60 a 69 anos	24 (33,3)	15 (62,5)		37,5 (19,0–56,0)		1,03 (0,38–2,85)	-
70 anos ou mais	15 (20,8)	11 (73,3)		26,7 (8,3–49,6)		1,34 (0,46–3,85)	-
Escolaridade			0,733		0,816		
9 anos ou mais	26 (36,1)	17 (65,4)		34,6 (17,5–52,5)		1,00	-
8 anos ou menos	42 (58,3)	24 (57,1)		42,9 (27,8–57,1)		0,82 (0,44–1,52)	-
Sem informação	4 (5,6)	2 (50,0)		50,0 (5,8–84,5)		0,85 (0,20–3,68)	-
Raça			0,629		0,355		
Branca	62 (86,1)	36 (58,1)		41,9 (29,6–53,8)		1,00	-
Outras	8 (11,1)	6 (75,0)		25,0 (3,7–55,8)		1,87 (0,78–4,45)	-
Sem informação	2 (2,8)	1 (50,0)		50,0 (0,6–91,0)		1,01 (0,14–7,34)	-
Situação conjugal			0,450		0,193		
Com companheiro	46 (63,9)	25 (54,4)		45,7 (31,0–59,2)		1,00	-
Sem companheiro	22 (30,6)	15 (68,2)		31,8 (14,2–51,1)		1,49 (0,78–2,82)	-
Sem informação	4 (5,6)	3 (75,0)		25,0 (0,9–66,5)		2,55 (0,76–8,50)	-
Extensão da doença			0,573		0,492		
Localizado	11 (15,3)	5 (45,5)		54,6 (22,9–78,0)		1,00	-
Metastático	43 (59,7)	27 (62,8)		37,2 (23,1–51,3)		1,77 (0,68–4,59)	-
Sem informação	18 (25,0)	11 (61,1)		38,9 (17,5–60,0)		1,70 (0,59–4,89)	-
Ano do diagnóstico			0,324		0,628		
2008	14 (19,4)	11 (78,6)		21,4 (5,2–44,8)		1,00	-
2009	21 (29,2)	12 (57,1)		42,9 (21,9–62,3)		0,67 (0,30–1,53)	-
2010	11 (15,3)	7 (63,6)		36,4 (11,2–62,7)		0,76 (0,29–1,95)	-
2011	13 (18,1)	5 (38,5)		61,5 (30,8–81,8)		0,36 (0,12–1,04)	-
2012	13 (18,1)	8 (61,5)		38,5 (14,1–62,8)		0,67 (0,27–1,67)	-
**Câncer de nasofaringe (C11)**							
Sexo			0,386		0,499		
Feminino	8 (33,3)	3 (37,5)		62,5 (22,9–86,1)		1,00	-
Masculino	16 (66,7)	9 (56,3)		43,8 (19,8–65,6)		1,56 (0,42–5,91)	-
Faixa etária			0,050		0,062		
65 anos ou menos	16 (66,7)	8 (50,0)			100,0	1,00	-
66 anos ou mais	8 (33,3)	8 (50,0)		25,0 (0,9–66,5)		1,29 (0,39–4,29)	-
Escolaridade			1,000		0,996		
9 anos ou mais	12 (50,0)	6 (50,0)		50,0 (20,9–73,6)		1,00	-
8 anos ou menos	10 (41,7)	5 (50,0)		50,0 (18,4–75,3)		1,06 (0,32–3,46)	-
Sem informação	2 (8,3)	1 (50,0)		50,0 (0,6–91,0)		1,01 (0,12–8,45)	-
Raça			0,307		0,398		
Branca	23 (95,8)	12 (52,2)		47,8 (26,8–66,1)		-	
Outras	1 (4,2)	-		-		-	
Situação conjugal			0,231		0,312		
Com companheiro	12 (50,0)	4 (33,3)		66,7 (33,7–86,0)		1,00	-
Sem companheiro	10 (41,7)	7 (70,0)		30,0 (7,1–57,8)		2,52 (0,74–8,64)	-
Sem informação	2 (8,3)	1 (50,0)		50,0 (0,6–91,0)		1,80 (0,18–14,42)	-
Extensão da doença			0,865		0,869		
Localizado	5 (20,8)	2 (40,0)		60,0 (12,6–88,2)		1,00	-
Metastático	11 (45,8)	6 (54,6)		45,5 (16,7–70,7)		1,54 (0,31–7,66)	-
Sem informação	8 (33,4)	4 (50,0)		50,0 (15,2–77,5)		1,34 (0,25–7,35)	-
Ano do diagnóstico			0,525		0,601		
2008	6 (25,0)	4 (66,7)		33,3 (4,6–67,6)		1,00	-
2009	5 (20,8)	3 (60,0)		40,0 (5,2–75,3)		0,85 (0,19–3,8)	-
2010	8 (33,3)	4 (50,0)		50,0 (15,2–77,5)		0,54 (0,14–2,18)	-
2011	2 (8,3)	-		100,0		-	-
2012	3 (12,5)	1 (33,3)		66,7 (5,4–94,5)		0,44 (0,05–3,99)	-
**Câncer de laringe (C32)**							
Sexo			0,385		0,352		
Feminino	16 (18,2)	5 (31,3)		68,8 (40,5–85,6)		1,00	-
Masculino	72 (81,8)	31 (43,1)		56,9 (44,7–67,4)		1,56 (0,61–4,01)	-
Faixa etária			0,430		0,434		
49 anos ou menos	11 (12,5)	2 (18,2)		81,8 (44,7–95,1)		1,00	-
50 a 59 anos	33 (37,5)	14 (42,4)		57,6 (39,1–72,3)		2,94 (0,67–12,95)	-
60 a 69 anos	22 (25,0)	10 (45,5)		54,6 (32,1–72,4)		3,23 (0,71–14,73)	-
70 anos ou mais	22 (25,0)	10 (45,5)		54,6 (32,1–72,4)		3,18 (0,70–14,54)	-
Escolaridade			0,529		0,599		
9 anos ou mais	43 (48,9)	15 (34,9)		65,1 (49,0–77,3)		1,00	-
8 anos ou menos	34 (38,6)	16 (47,1)		52,9 (35,1–68,0)		1,47 (0,73–2,98)	-
Sem informação	11 (12,5)	5 (45,5)		54,6 (22,9–78,0)		-	-
Raça			0,529		0,974		
Branca	78 (88,6)	32 (41,0)		59,0 (47,3–68,9)		1,00	-
Outras	7 (8,0)	3 (42,9)		57,1 (17,2–83,7)		1,44 (0,44–4,73)	-
Sem informação	3 (3,4)	1 (33,3)		66,7 (5,4–94,5)		-	-
Situação conjugal			0,924		0,924		
Com companheiro	54 (61,4)	22 (40,7)		59,3 (45,0–71,0)		1,00	-
Sem companheiro	23 (26,1)	10 (43,5)		56,5 (34,3–73,8)		1,04 (0,49–2,22)	-
Sem informação	11 (12,5)	4 (36,4)		63,6 (29,7–84,5)		-	-
Extensão da doença			0,005		0,004		
Localizado	31 (35,2)	7 (22,6)		77,4 (58,4–88,5)		1,00	-
Metastático	28 (31,8)	18 (64,3)		35,7 (18,9–53,0)		3,79 (1,58–9,08)	-
Sem informação	29 (33,0)	11 (40,9)		62,1 (42,1–76,9)		1,77 (0,69–4,57)	-
Ano do diagnóstico			0,337		0,383		
2008	15 (17,1)	8 (53,3)		46,7 (21,2–68,8)		1,00	-
2009	16 (18,2)	5 (31,3)		68,8 (40,5–85,6)		0,50 (0,16–1,54)	-
2010	22 (25,0)	11 (50,0)		50,0 (28,2–68,4)		0,83 (0,33–2,06)	-
2011	18 (20,5)	8 (44,4)		55,6 (30,5–74,8)		0,65 (0,24–1,72)	-
2012	17 (19,3)	4 (23,5)		76,5 (48,8–90,5)		0,34 (0,10–1,14)	-
**Câncer de traqueia, brônquios e pulmão (C33-C34)**^**d**^							
Sexo			0,038		0,044		
Feminino	199 (37,8)	144 (72,4)		27,6 (21,6–34,0)		1,00	1,00
Masculino	328 (62,2)	263 (80,2)		19,8 (15,7–24,3)		1,23 (1,01–1,51)	1,19 (0,97–1,46)
Faixa etária			0,080		0,018		
49 anos ou menos	42 (8,0)	31 (73,8)		26,2 (14,1–40,0)		1,00	1,00
50 a 59 anos	117 (22,2)	81 (69,2)		30,8 (22,7–39,2)		0,96 (0,63–1,45)	0,96 (0,63–1,46)
60 a 69 anos	166 (31,6)	132 (79,5)		20,5 (14,7–26,9)		1,27 (0,86–1,88)	1,18 (0,80–1,75)
70 anos ou mais	201 (38,2)	163 (81,1)		18,9 (13,8–24,6)		1,42 (0,97–2,09)	1,34 (0,92–1,98)
Escolaridade			0,258		0,013		
9 anos ou mais	241 (45,7)	179 (74,3)		25,7 (20,4–31,4)		1,00	1,00
8 anos ou menos	227 (43,1)	183 (80,6)		19,4 (14,5–24,8)		1,36 (1,11–1,67)	1,29 (1,05–1,59)
Sem informação	59 (11,2)	45 (77,2)		23,7 (13,9–35,1)		1,10 (0,79–1,52)	1,29 (0,93–1,81)
Raça			0,617		0,445		
Branca	478 (90,7)	368 (77,0)		23,0 (19,4–26,9)		1,00	-
Outras	26 (4,9)	22 (84,6)		15,4 (4,8–31,5)		1,29 (0,84–1,98)	-
Sem informação	23 (4,4)	17 (73,9)		26,1 (10,6–44,7)		0,89 (0,55–1,45)	-
Situação conjugal			0,637		0,692		
Com companheiro	309 (58,6)	243 (78,6)		21,4 (17,0–26,1)		1,00	-
Sem companheiro	168 (31,9)	127 (75,6)		24,4 (18,2–31,1)		0,99 (0,8–1,23)	-
Sem informação	50 (9,5)	37 (74,0)		26,0 (14,9–38,6)		0,86 (0,61–1,22)	-
Extensão da doença			< 0,001		< 0,001		
Localizado	60 (11,4)	36 (60,0)		40,0 (27,7–52,0)		1,00	1,00
Metastático	302 (57,3)	254 (84,1)		15,9 (12,0–20,3)		2,06 (1,45–2,92)	2,14 (1,40–2,85)
Sem informação	165 (31,3)	117 (70,9)		29,1 (22,4–36,1)		1,34 (0,92–1,94)	1,38 (0,90–1,91)
Ano do diagnóstico			0,487		0,503		
2008	93 (17,7)	72 (77,4)		22,6 (14,7–31,5)		1,00	-
2009	112 (21,3)	92 (82,1)		17,9 (11,4–25,5)		1,17 (0,86–1,59)	-
2010	105 (19,9)	77 (73,3)		26,7 (18,6–35,4)		0,93 (0,68–1,29)	-
2011	102 (19,4)	81 (79,4)		20,6 (13,4–28,9)		1,18 (0,86–1,62)	-
2012	115 (21,8)	85 (73,9)		26,1 (18,5–34,3)		1,03 (0,76–1,42)	-

S(t): probabilidade de sobrevivência em função do tempo; IC95%: intervalo de confiança de 95%; HR: razão de riscos proporcionais

^a^ Valor de p do teste qui-quadrado.

^b^ Valor de p do teste Log-rank.

^c^ HR ajustada por faixa etária, raça, escolaridade e extensão de doença.

^d^ HR ajustada por sexo, faixa etária, escolaridade e extensão de doença.

### Outras faringes (C09-10; C12-14)

Foram registrados 72 casos referentes a outras faringes, ou 2,6% do total. Desses, 59,7% foram a óbito até o fim do acompanhamento e a probabilidade de sobrevivência foi de 40,3% (IC95% 29,0–51,3) (Tabela 1 e Figura 1). Foi mais frequente no sexo masculino, com menor escolaridade e naqueles com companheiro. Nenhuma variável se mostrou associada à sobrevivência e não foi realizada análise ajustada (Tabela 2).

### Nasofaringe (C11)

Diagnósticos de nasofaringe representaram 0,9%, com 24 casos. Desses, 50% foram a óbito e a probabilidade de sobrevivência foi de 50,0% (IC95% 29,1–67,8) (Tabela 1 e Figura 1). Houve mais registros em homens, com 65 anos ou menos, e nove anos ou mais de escolaridade. Apenas a variável faixa etária apresentou valor de p < 0,20, no qual o grupo com 66 anos ou mais demonstrou pior probabilidade de sobrevivência (Tabela 2).

### Esôfago (C15)

No período, houve 104 registros de câncer de esôfago, ou 3,7% do total. Foram a óbito 67,6% e a probabilidade de sobrevivência foi de 32,4% (IC95% 23,7–41,1) (Tabela 1 e Figura 1). A pior sobrevivência foi encontrada entre aqueles do sexo masculino, de outras raças/cores, sem companheiro, com diagnóstico metastático. A análise ajustada incluiu as variáveis sexo, raça, situação conjugal, extensão da doença e ano do diagnóstico. Apenas outras cores/raças agrupadas se mantiveram como fator de pior prognóstico, com aumento de 2,5 no risco de óbito (Tabela 3).

**Tabela 3:** Descrição, sobrevivência e riscos proporcionais de óbito dos casos registrados de cânceres esôfago (C15), estômago (C16), colón e reto (C18-21), fígado (C22) e pâncreas (C25), Florianópolis, 2008–2017.

Variáveis	n (%)	Óbitos	p^a^	S(t) (IC95%)	p^b^	HR bruta (IC95%)	HR ajustada (IC95%)

		n (%)					
**Câncer de esôfago (C15)**^**c**^							
Sexo			0,140		0,127		
Feminino	22 (21,0)	12 (54,6)		45,5 (24,4–64,3)		1,00	1,00
Masculino	83 (79,1)	59 (71,1)		28,9 (19,6–38,9)		1,62 (0,87–3,01)	1,64 (0,82–3,28)
Faixa etária			0,577		0,351		
49 anos ou menos	9 (8,6)	7 (77,8)		22,2 (3,4–51,3)		1,00	-
50 a 59 anos	39 (37,1)	28 (71,8)		28,2 (15,3–42,7)		0,64 (0,28–1,48)	-
60 a 69 anos	24 (32,4)	20 (58,8)		41,2 (24,8–56,9)		0,47 (0,20–1,12)	-
70 anos ou mais	23 (21,9)	16 (69,6)		30,4 (13,5–49,3)		0,67 (0,28–1,64)	-
Escolaridade			0,457		0,486		
9 anos ou mais	31 (29,5)	21 (67,7)		32,3 (16,9–48,6)		1,00	-
8 anos ou menos	56 (53,3)	40 (71,4)		28,6 (17,5–40,7)		1,34 (0,79–2,27)	-
Sem informação	18 (17,1)	10 (55,6)		44,4 (21,6–65,1)		1,01 (0,47–2,14)	-
Raça			0,015		0,002		
Branca	83 (79,1)	54 (65,1)		34,9 (24,9–45,1)		1,00	1,00
Outras	16 (15,2)	15 (93,8)		6,3 (0,4–24,7)		2,54 (1,42–4,56)	2,50 (1,27–4,90)
Sem informação	6 (5,7)	2 (33,3)		66,7 (19,5–90,4)		0,43 (0,10–1,76)	0,57 (0,09–3,35)
Situação conjugal			0,073		0,074		
Com companheiro	64 (61,0)	44 (68,8)		31,3 (20,4–42,7)		1,00	1,00
Sem companheiro	27 (25,7)	21 (77,8)		22,2 (9,0–39,0)		1,52 (0,90–2,56)	1,39 (0,80–2,43)
Sem informação	14 (13,3)	6 (42,9)		57,1 (28,4–78,0)		0,57 (0,24–1,35)	1,26 (0,38–4,17)
Extensão da doença			0,009		0,046		
Localizado	24 (22,9)	13 (54,2)		45,8 (25,6–64,0)		1,00	1,00
Metastático	37 (35,2)	32 (86,5)		13,5 (4,9–26,4)		1,96 (1,02–3,74)	2,09 (0,91–4,82)
Sem informação	44 (41,9)	26 (59,1)		40,9 (26,5–54,8)		1,14 (0,59–2,22)	1,31 (0,58–2,96)
Ano do diagnóstico			0,012		0,021		
2008	20 (19,1)	9 (45,0)		55,0 (31,3–73,5)		1,00	1,00
2009	18 (17,1)	17 (94,4)		5,6 (0,4–22,4)		3,43 (1,51–7,82)	2,61 (0,96–7,05)
2010	18 (17,1)	10 (55,6)		44,4 (21,6–65,1)		1,32 (0,54–3,25)	1,25 (0,44–3,57)
2011	15 (14,3)	12 (80,0)		20,0 (4,9–42,4)		2,16 (0,91–5,15)	1,67 (0,56–4,94)
2012	34 (32,4)	23 (67,7)		32,4 (17,6–48)		1,72 (0,79–3,71)	1,23 (0,45–3,35)
**Câncer de estômago (C16)**^**d**^							
Sexo			0,099		0,094		
Feminino	142 (42,6)	78 (54,9)		45,1 (36,8–53,0)		1,00	1,00
Masculino	191 (57,4)	122 (63,9)		36,1 (29,4–42,9)		1,27 (0,96–1,69)	1,21 (0,91–1,61)
Faixa etária			< 0,001		< 0,001		
59 anos ou menos	125 (37,5)	54 (43,2)		56,8 (47,7–64,9)		1,00	1,00
60 anos ou mais	208 (62,5)	146 (70,2)		29,8 (23,7–36,1)		2,18 (1,59–2,98)	2,22 (1,62–3,03)
Escolaridade			0,214		0,229		
9 anos ou mais	121 (36,3)	71 (58,7)		41,3 (32,5–49,9)		1,00	-
8 anos ou menos	182 (54,7)	115 (63,2)		36,8 (29,9–43,8)		1,18 (0,88–1,59)	-
Sem informação	30 (9,0)	14 (46,7)		53,3 (34,3–69,1)		0,78 (0,44–1,38)	-
Raça			0,229		0,277		
Branca	293 (88,0)	177 (60,4)		39,6 (34,0–45,1)		1,00	-
Outras	29 (8,7)	19 (65,5)		34,5 (18,2–51,5)		1,18 (0,74–1,90)	-
Sem informação	11 (3,3)	4 (36,4)		63,6 (29,7–84,5)		0,50 (0,18–1,34)	-
Situação conjugal			0,195		0,215		
Com companheiro	209 (62,8)	124 (59,3)		40,7 (34,0–47,2)		1,00	-
Sem companheiro	98 (29,4)	64 (65,3)		34,7 (25,5–44,1)		1,16 (0,86–1,57)	-
Sem informação	26 (7,8)	12 (46,2)		53,9 (33,3–70,6)		0,21 (0,38–1,24)	-
Extensão da doença			< 0,001		0,002		
Localizado	54 (16,2)	20 (37)		63,0 (48,7–74,3)		1,00	1,00
Metastático	135 (40,5)	93 (68,9)		31,1 (23,5–39,0)		2,31 (1,42–3,75)	2,39 (1,46–3,88)
Sem informação	144 (43,2)	87 (60,4)		39,6 (31,6–47,5)		2,00 (1,23–3,25)	1,92 (1,18–3,13)
Ano do diagnóstico			0,594		0,714		
2008	55 (16,5)	36 (65,5)		34,6 (22,4–47,0)		1,00	-
2009	54 (16,2)	34 (63,0)		37,0 (24,4–49,7)		0,95 (0,59–1,52)	-
2010	77 (23,1)	42 (54,6)		45,5 (34,1–56,1)		0,76 (0,49–1,19)	-
2011	75 (22,5)	42 (56)		44,0 (32,6–54,8)		0,80 (0,51–1,25)	-
2012	72 (21,6)	46 (63,9)		36,1 (25,2–47,1)		0,91 (0,59–1,40)	-
**Câncer de cólon e reto (C18-21)**^**e**^							
Sexo			0,742		0,906		
Feminino	403 (49,6)	142 (35,2)		64,8 (59,9–69,2)		1,00	-
Masculino	410 (50,4)	149 (36,3)		63,7 (58,8–68,1)		1,01 (0,81–1,28)	-
Faixa etária			< 0,001		< 0,001		
49 anos ou menos	157 (19,3)	41 (26,1)		73,9 (66,3–80,0)		1,00	1,00
50 a 59 anos	186 (22,9)	50 (26,9)		73,1 (66,1–78,9)		1,01 (0,67–1,53)	1,17 (0,77–1,78)
60 a 69 anos	206 (25,3)	76 (36,9)		63,1 (56,1–69,3)		1,48 (1,02–2,17)	1,61 (1,10–2,37)
70 anos ou mais	264 (32,5)	124 (47,0)		53,0 (46,8–58,8)		2,15 (1,51–3,05)	2,54 (1,77–3,65)
Escolaridade			< 0,001		< 0,001		
9 anos ou mais	443 (54,5)	150 (33,9)		66,1 (61,5–70,3)		1,00	1,00
8 anos ou menos	254 (31,2)	117 (46,1)		53,9 (47,6–59,8)		1,48 (1,17–1,89)	1,29 (1,01–1,66)
Sem informação	116 (14,3)	24 (20,7)		79,3 (70,7–85,6)		0,58 (0,38–0,89)	0,64 (0,32–1,29)
Raça			0,028		0,045		
Branca	703 (86,5)	262 (37,3)		62,7 (59,0–66,2)		1,00	1,00
Outras	43 (5,3)	15 (34,9)		65,1 (49,0–77,3)		0,95 (0,56–1,60)	1,05 (0,62–1,80)
Sem informação	67 (8,2)	14 (20,9)		79,1 (67,3–87,1)		0,51 (0,30–0,88)	1,32 (0,60–2,91)
Situação conjugal			< 0,001		< 0,001		
Com companheiro	436 (53,6)	150 (34,4)		65,6 (60,9–69,9)		1,00	1,00
Sem companheiro	268 (33)	117 (43,7)		56,3 (50,2–62,0)		1,41 (1,11–1,80)	1,33 (1,05–1,71)
Sem informação	109 (13,4)	24 (22,0)		78,0 (69,0–84,7)		0,62 (0,40–0,95)	0,80 (0,38–1,66)
Extensão da doença			< 0,001		< 0,001		
In situ	31 (3,8)	5 (16,1)		83,9 (65,5–93)		1,00	1,00
Localizado	175 (21,5)	39 (22,3)		77,7 (70,8–83,2)		1,42 (0,56–3,61)	1,23 (0,49–3,16)
Metastático	297 (36,5)	158 (53,2)		46,8 (41,0–52,4)		4,13 (1,69–10,06)	4,74 (1,92–11,66)
Sem informação	310 (38,1)	89 (28,7)		71,3 (65,9–76)		1,94 (0,79–4,78)	1,95 (0,78–4,83)
Ano do diagnóstico			0,008		0,005		
2008	136 (16,7)	48 (35,3)		64,7 (56,1–72,1)		1,00	1,00
2009	176 (21,7)	55 (31,3)		68,8 (61,3–75,0)		0,85 (0,58–1,25)	0,50 (0,33–0,75)
2010	165 (20,3)	73 (44,2)		55,8 (47,9–63,0)		1,37 (0,95–1,97)	0,79 (0,53–1,18)
2011	148 (18,2)	62 (41,9)		58,1 (49,7–65,6)		1,20 (0,82–1,75)	0,55 (0,36–0,84)
2012	188 (23,1)	53 (28,2)		71,8 (64,8–77,7)		0,75 (0,51–1,11)	0,38 (0,25–0,59)
**Câncer de fígado (C22)**^**f**^							
Sexo			0,727		0,407		
Feminino	29 (28,2)	24 (82,8)		17,2 (6,3–32,7)		1,00	-
Masculino	74 (71,8)	59 (79,7)		20,3 (12,0–30,0)		0,82 (0,51–1,32)	-
Faixa etária			0,022		0,182		
49 anos ou menos	18 (17,5)	10 (55,6)		44,4 (21,6–65,1)		1,00	1,00
50 a 59 anos	25 (24,3)	23 (92,0)		8,0 (1,4–22,5)		2,04 (0,97–4,31)	1,84 (0,86–3,94)
60 a 69 anos	23 (22,3)	19 (82,6)		17,4 (5,4–35,0)		1,79 (0,83–3,86)	1,77 (0,82–3,82)
70 anos ou mais	37 (35,9)	31 (83,8)		16,2 (6,6–29,6)		2,14 (1,05–4,38)	2,19 (1,06–4,53)
Escolaridade			0,579		0,678		
9 anos ou mais	53 (51,4)	41 (77,4)		22,6 (12,5–34,6)		1,00	-
8 anos ou menos	36 (35,0)	31 (86,1)		13,9 (5,1–27,1)		1,23 (0,77–1,96)	-
Sem informação	14 (13,6)	11 (78,6)		21,4 (5,2–44,8)		1,07 (0,55–2,09)	-
Raça			0,606		0,010		
Branca	94 (91,3)	75 (79,8)		20,2 (12,8–28,8)		1,00	1,00
Outras	4 (3,8)	4 (100,0)		–		4,33 (1,53–12,26)	4,11 (1,40–12,07)
Sem informação	5 (4,9)	4 (80,0)		20 (0,8–58,2)		1,07 (0,39–2,94)	0,85 (0,30–2,44)
Situação conjugal			0,529		0,507		
Com companheiro	54 (52,4)	44 (81,5)		18,5 (9,5–29,8)		1,00	-
Sem companheiro	36 (35,0)	30 (83,3)		16,7 (6,8–30,4)		1,15 (0,72–1,83)	-
Sem informação	13 (12,6)	9 (69,2)		30,8 (9,5–55,4)		0,74 (0,36–1,52)	-
Extensão da doença			0,284		0,746		
Localizado	11 (10,7)	7 (63,6)		36,4 (11,2–62,7)		1,00	-
Metastático	40 (38,8)	34 (85,0)		15,0 (6,1–27,6)		1,33 (0,59–3,00)	-
Sem informação	52 (50,5)	42 (80,8)		19,2 (9,9–30,9)		1,36 (0,61–3,03)	-
Ano do diagnóstico			0,212		0,203		
2008	25 (24,2)	18 (72,0)		28,0 (12,4–46)		1,00	-
2009	19 (18,4)	14 (73,7)		26,3 (9,6–46,8)		1,01 (0,50–2,03)	-
2010	22 (21,4)	21 (95,5)		4,6 (0,3–18,9)		1,84 (0,98–3,47)	-
2011	15 (14,6)	11 (73,3)		26,7 (8,3–49,6)		1,05 (0,50–2,23)	-
2012	22 (21,4)	19 (86,4)		13,6 (3,4–30,9)		1,57 (0,82–3,00)	-
**Câncer de pâncreas (C25)**^**f**^							
Sexo			0,642		0,293		
Feminino	62 (55,4)	54 (87,1)		12,9 (6,0–22,5)		1,00	
Masculino	50 (44,6)	42 (85,0)		16,0 (7,5–27,4)		0,81 (0,54–1,21)	
Faixa etária			0,340		0,072		
49 anos ou menos	10 (8,9)	7 (70,0)		30,0 (7,1–57,8)		1,00	1,00
50 a 59 anos	24 (21,4)	20 (83,3)		16,7 (5,2–33,7)		1,30 (0,55–3,08)	1,19 (0,49–2,87)
60 a 69 anos	24 (21,4)	20 (83,3)		16,7 (5,2–33,7)		1,21 (0,51–2,85)	1,19 (0,50–2,82)
70 anos ou mais	54 (48,2)	49 (90,7)		9,3 (3,4–18,7)		2,05 (0,93–4,54)	1,91 (0,85–4,29)
Escolaridade			0,057		0,467		
9 anos ou mais	50 (44,6)	46 (92,0)		8,0 (2,6–17,5)		1,00	-
8 anos ou menos	54 (48,2)	42 (77,8)		22,2 (12,3–34)		0,79 (0,52–1,20)	-
Sem informação	8 (7,2)	8 (100,0)		–		0,73 (0,34–1,54)	-
Raça			0,236		0,638		
Branca	97 (86,6)	81 (83,5)		16,5 (9,9–24,5)		1,00	-
Outras	10 (8,9)	10 (100,0)		–		1,35 (0,70–2,62)	-
Sem informação	5 (4,5)	5 (100,0)		–		0,89 (0,36–2,21)	-
Situação conjugal			0,987		0,623		
Com companheiro	65 (58,0)	56 (86,2)		13,9 (6,8–23,3)		1,00	-
Sem companheiro	40 (35,7)	34 (85,0)		15,0 (6,1–27,6)		1,23 (0,80–1,88)	-
Sem informação	7 (6,3)	6 (85,7)		14,3 (0,7–46,5)		0,97 (0,42–2,24)	-
Extensão da doença			0,638		0,172		
Localizado	8 (7,1)	7 (87,5)		12,5 (0,7–42,3)		1,00	1,00
Metastático	73 (65,2)	64 (87,7)		12,3 (6,1–21,0)		1,75 (0,80–3,82)	1,48 (0,67–3,45)
Sem informação	31 (27,7)	25 (80,7)		19,4 (7,9–34,6)		1,24 (0,54–2,87)	0,87 (0,45–2,59)
Ano do diagnóstico			0,764		0,885		
2008	14 (12,5)	12 (85,7)		14,3 (2,3–36,6)		1,00	-
2009	21 (18,8)	19 (90,5)		9,5 (1,6–26,1)		1,15 (0,56–2,37)	-
2010	28 (25,0)	25 (89,3)		10,7 (2,7–25,1)		1,09 (0,55–2,17)	-
2011	28 (25,0)	22 (78,6)		21,4 (8,7–37,8)		0,85 (0,42–1,72)	-
2012	21 (18,8)	18 (85,7)		14,3 (3,6–32,1)		1,06 (0,51–2,21)	-

S(t): probabilidade de sobrevivência em função do tempo; IC95%: intervalo de confiança de 95%; HR: razão de riscos proporcionais.

^a^ Valor de p do teste qui-quadrado.

^b^ Valor de p do teste Log-rank.

^c^ HR ajustada por sexo, raça, situação conjugal, extensão da doença e ano do diagnóstico.

^d^ HR ajustada por sexo, faixa etária e extensão da doença.

^e^ HR ajustada por faixa etária, raça, situação conjugal, extensão da doença e ano do diagnóstico.

^f^ HR ajustada por faixa etária e extensão da doença.

### Estômago (C16)

O câncer de estômago foi o terceiro mais frequente, com 333 casos (11,8% do total). Desses, 60,1% foram a óbito, e a probabilidade de sobrevivência foi de 39,9% (IC95% 34,7–45,2) (Tabela 1 e Figura 1). As piores probabilidades de sobrevivência estiveram associadas a ter 60 anos ou mais e diagnóstico metastático. As variáveis sexo, faixa etária e extensão da doença foram incluídas na análise ajustada. O risco de óbito foi aumentado de forma independente entre os com 60 anos ou mais e naqueles com diagnóstico metastático ou registrados como sem informação da extensão da doença (Tabela 3).

### Colón e reto (C18-21)

O câncer de cólon e reto foi o mais frequente, com 813 registros (28,7% do total), dos quais 35,8% foram a óbito. A probabilidade de sobrevivência em cinco anos foi de 64,2% (IC95% 60,8–67,4) (Tabela 1 e Figura 1). As piores taxas de sobrevivência foram entre as pessoas com 70 anos ou mais, com nove anos ou mais de escolaridade, da raça/cor branca e com diagnóstico metastático. Na análise ajustada, ter acima de 60 anos, oito anos ou menos de escolaridade, não ter companheiro e ter diagnóstico metastático aumentaram de forma independente o risco de óbito (Tabela 3).

### Fígado (C22)

O câncer de fígado teve 103 registros (3,6% do total), dos quais 80,6% foram a óbito. Esse câncer foi mais frequente em homens, pessoas brancas e com companheiro. A probabilidade de sobrevivência em cinco anos foi de 19,4% (IC95% 12,5–27,6), a segunda mais baixa (Tabela 1 e Figura 1). Essa foi associada à faixa etária e à raça. Na análise ajustada, ter 70 anos ou mais aumentou em 2,19 o risco de óbito, enquanto estar na categoria outra cor/raça, aumentou em 4 vezes (Tabela 3).

### Pâncreas (C25)

O câncer de pâncreas teve 112 casos registrados (4,0% do total) e no acompanhamento, 85,7% foram a óbito. Apresentou a pior probabilidade de sobrevivência em cinco anos, com 14,3% (IC95% 8,6–21,4) (Tabela 1 e Figura 1). As probabilidades de sobrevivência foram baixas em todas as categorias de variáveis analisadas. Foram associadas à sobrevivência a faixa etária e a extensão da doença. Essas variáveis foram incluídas na análise ajustada, mas nenhuma aumentou de forma independente o risco de óbito (Tabela 3).

### Laringe (C32)

O câncer de laringe foi registrado em 88 pessoas (3,1% do total). No acompanhamento, 40,9% foram a óbito, e a probabilidade de sobrevivência em cinco anos foi de 59,1% (IC95% 48,1–68,5) (Tabela 1 e Figura 1). Houve maior número de registros em homens, de 50 a 59 anos, com nove anos ou mais de escolaridade e com companheiro. Nenhuma variável foi associada à sobrevivência (Tabela 2).

### Traqueia, Brônquios e Pulmão (C33-C34)

O câncer de traqueia, brônquios e pulmão foi o terceiro mais frequente, com 527 casos (18,6% do total). No acompanhamento, 77,2% foram a óbito e a probabilidade de sobrevivência em cinco anos foi a terceira pior, 22,8% (IC95% 19,3–26,4) (Tabela 1 e Figura 1). O diagnóstico foi mais frequente em homens, com mais de 60 anos, raça branca, com companheiro e extensão metastática. As variáveis sexo, faixa etária, escolaridade e extensão da doença foram adicionadas na análise ajustada. Os fatores de risco independentes para o óbito foram ter oito anos ou menos escolaridade (aumento de 18%) e o estadiamento metastático (incremento de 2,14) (Tabela 2).

### Rim (C64-C66)

O câncer de rim teve 177 registros (6,3% do total). Foram a óbito, em cinco anos, 24,3% da amostra, e a probabilidade de sobrevivência no mesmo período foi de 75,7% (IC95% 68,7–81,4) (Tabela 1 e Figura 1). Foi mais frequente em homens, de raça branca e com companheiro. As piores taxas de sobrevivência foram registradas entre aqueles com 70 anos ou mais, de outras raças/cor, sem companheiro e com diagnóstico metastático. Na análise ajustada para faixa etária, escolaridade, raça, situação conjugal e extensão da doença, foram fatores de risco independentes para o óbito: ter idade de 70 anos ou mais (HR 3,77; IC95% 1,01–14,06) e ser da raça branca (HR 3,69; IC95% 1,20–11,29) (Tabela 4).

**Tabela 4:** Descrição, sobrevivência e riscos proporcionais de óbito dos casos registrados de cânceres de rim (C64-C66), bexiga (C67) e leucemia mieloide aguda (C92.0), Florianópolis, 2008–2017.

Variáveis	n (%)	Óbitos	p^a^	S(t) (IC95%)	p^b^	HR bruta (IC95%)	HR ajustada (IC95%)

		n (%)					
**Câncer de Rim (C64-C66)**^**c**^							
Sexo			0,421		0,441		
Feminino	71 (40,1)	15 (21,1)		78,9 (67,4–86,7)		1,00	-
Masculino	106 (59,9)	28 (26,4)		73,6 (64,1–80,9)		1,28 (0,68–2,39)	-
Faixa etária			0,011		0,010		
49 anos ou menos	31 (17,5)	3 (9,7)		90,3 (72,9–96,8)		1,00	1,00
50 a 59 anos	51 (28,8)	9 (17,7)		82,4 (68,8–90,4)		1,89 (0,51–6,99)	1,54 (0,40–5,96)
60 a 69 anos	62 (35,0)	17 (27,4)		72,6 (59,7–82,0)		3,12 (0,92–10,66)	2,57 (0,73–9,12)
70 anos ou mais	33 (18,6)	14 (42,4)		57,6 (39,1–72,3)		5,33 (1,53–18,55)	3,77 (1,01–14,06)
Escolaridade			0,129		0,158		
9 anos ou mais	91 (51,4)	24 (26,4)		73,6 (63,3–81,5)		1,00	1,00
8 anos ou menos	51 (28,8)	15 (29,4)		70,6 (56,0–81,1)		1,19 (0,62–2,26)	1,06 (0,54–2,07)
Sem informação	35 (19,8)	4 (11,4)		88,6 (72,4–95,6)		0,42 (0,15–1,21)	0,94 (0,20–4,48)
Raça			0,023		0,020		
Branca	152 (85,8)	38 (25,0)		75,0 (67,3–81,1)		1,00	1,00
Outras	7 (4,0)	4 (57,1)		42,9 (9,8–73,4)		2,77 (0,99–7,78)	3,69 (1,20–11,29)
Sem informação	18 (10,2)	1 (5,6)		94,4 (66,6–99,2)		0,20 (0,03–1,49)	0,40 (0,04–3,60)
Situação conjugal			0,061		0,073		
Com companheiro	92 (52,0)	23 (25,0)		75,0 (64,8–82,6)		1,00	1,00
Sem companheiro	44 (24,9)	15 (34,1)		65,9 (50,0–77,9)		1,45 (0,76–2,78)	1,57 (0,80–3,12)
Sem informação	41 (23,8)	5 (12,2)		87,8 (73,2–94,7)		0,47 (0,18–1,23)	0,96 (0,24–3,87)
Extensão da doença			< 0,001		< 0,001		
Localizado	33 (18,6)	6 (18,2)		81,8 (63,9–91,4)		1,00	1,00
Metastático	45 (25,4)	22 (48,9)		51,1 (35,8–64,5)		3,36 (1,36–8,29)	1,99 (0,75–5,29)
Sem informação	99 (55,9)	15 (15,2)		84,9 (76,1–90,6)		0,81 (0,31–2,07)	0,57 (0,20–1,56)
Ano do diagnóstico			0,956		0,969		
2008	37 (20,9)	8 (21,6)		78,4 (61,4–88,6)		1,00	-
2009	34 (19,2)	9 (26,5)		73,5 (55,3–85,3)		1,31 (0,50–3,39)	-
2010	49 (27,7)	13 (26,5)		73,5 (58,7–83,6)		1,22 (0,51–2,95)	-
2011	34 (19,2)	7 (20,6)		79,4 (61,6–89,6)		0,99 (0,36–2,73)	-
2012	23 (13,0)	6 (26,1)		73,9 (50,9–87,3)		1,24 (0,43–3,56)	-
**Câncer de Bexiga (C67)**^**d**^							
Sexo			0,938		0,987		
Feminino	73 (25,9)	22 (30,1)		69,9 (57,9–79,0)		1,00	-
Masculino	209 (74,1)	64 (30,6)		69,4 (62,6–75,1)		1,00 (0,62–1,63)	-
Faixa etária			0,001		< 0,002		
49 anos ou menos	25 (8,9)	2 (8,0)		92,0 (71,6–97,9)		1,00	1,00
50 a 59 anos	55 (19,5)	12 (21,8)		78,2 (64,8–87,0)		2,85 (0,64–12,75)	3,00 (0,66–13,52)
60 a 69 anos	71 (25,2)	17 (23,9)		76,1 (64,3–84,4)		3,21 (0,74–13,90)	3,25 (0,74–14,19)
70 anos ou mais	131 (46,5)	55 (42,0)		58,0 (49,1–65,9)		6,22 (1,52–25,51)	6,19 (1,49–25,82)
Escolaridade			0,131		0,134		
9 anos ou mais	115 (40,8)	35 (30,4)		69,6 (60,3–77,1)		1,00	1,00
8 anos ou menos	109 (38,7)	39 (35,8)		64,2 (54,5–72,4)		1,24 (0,79–1,96)	1,09 (0,68–1,73)
Sem informação	58 (20,6)	12 (20,7)		79,3 (66,5–87,7)		0,65 (0,34–1,26)	0,89 (0,36–2,19)
Raça			0,129		0,164		
Branca	237 (84,0)	78 (32,9)		67,1 (60,7–72,7)		1,00	1,00
Outras	12 (4,3)	2 (16,7)		83,3 (48,2–95,6)		0,47 (0,11–1,9)	0,65 (0,16–2,68)
Sem informação	33 (11,7)	6 (18,2)		81,8 (63,9–91,4)		0,51 (0,22–1,18)	0,85 (0,27–2,68)
Situação conjugal			0,368		0,256		
Com companheiro	151 (53,6)	45 (29,8)		70,2 (62,2–76,8)		1,00	-
Sem companheiro	78 (27,7)	28 (35,9)		64,1 (52,4–73,6)		1,36 (0,85–2,18)	-
Sem informação	53 (18,8)	13 (24,5)		75,5 (61,5–85,0)		0,83 (0,45–1,54)	-
Extensão da doença			< 0,001		< 0,001		
In situ	17 (6,0)	5 (29,4)		70,6 (43,2–86,6)		1,00	1,00
Localizado	95 (33,7)	19 (20,0)		80,0 (70,5–86,7)		0,66 (0,25–1,78)	0,59 (0,22–1,58)
Metastático	36 (12,8)	22 (61,1)		38,9 (23,3–54,2)		2,79 (1,06–7,37)	2,38 (0,89–6,33)
Sem informação	134 (47,5)	40 (29,9)		70,2 (61,6–77,1)		1,05 (0,41–2,66)	1,00 (0,39–2,56)
Ano do diagnóstico			0,567		0,605		
2008	67 (23,8)	16 (23,9)		76,1 (64,0–84,6)		1,00	-
2009	58 (20,6)	20 (34,5)		65,5 (51,8–76,2)		1,53 (0,79–2,96)	-
2010	54 (19,2)	18 (33,3)		66,7 (52,4–77,5)		1,44 (0,74–2,83)	-
2011	54 (19,2)	19 (35,2)		64,8 (50,6–75,9)		1,57 (0,81–3,05)	-
2012	49 (17,4)	13 (26,5)		73,5 (58,7–83,6)		1,13 (0,54–2,34)	-
**Leucemia mieloide aguda (C92.0)**							
Sexo			0,934		0,819		
Feminino	15 (53,6)	9 (60,0)		40,0 (16,5–62,8)		1,00	-
Masculino	13 (46,4)	8 (61,5)		38,5 (14,1–62,8)		1,12 (0,43–2,90)	-
Faixa etária			0,247		0,205		
65 anos ou menos	15 (55,6)	8 (53,3)		46,7 (21,2–68,8)		1,00	-
66 anos ou mais	12 (44,4)	9 (75,0)		25,0 (6,0–50,5)		1,84 (0,71–4,81)	-
Escolaridade			0,010		0,015		
9 anos ou mais	16 (57,1)	13 (81,3)		18,8 (4,6–40,3)		1,00	-
8 anos ou menos	7 (25,0)	1 (14,3)		85,7 (33,4–97,9)		0,09 (0,01–0,72)	-
Sem informação	5 (17,9)	3 (60,0)		40,0 (5,2–75,3)		0,45 (0,12–1,61)	-
Raça			0,444		0,432		
Branca	24 (85,7)	14 (62,5)		37,5 (19,0–56,0)		1,00	-
Outras	1 (3,6)	1 (100,0)		-		1,96 (0,25–15,37)	-
Sem informação	3 (10,7)	1 (33,3)		66,7 (5,4–94,5)		0,35 (0,05–2,64)	-
Situação conjugal			0,819		0,827		
Com companheiro	12 (42,9)	8 (66,7)		33,3 (10,3–58,8)		1,00	-
Sem companheiro	12 (42,9)	7 (58,3)		41,7 (15,3–66,5)		0,82 (0,30–2,27)	-
Sem informação	4 (14,3)	2 (50,0)		50,0 (5,8–84,5)		0,63 (0,13–3,01)	-
Ano do diagnóstico			0,584		0,714		
2008	4 (14,3)	2 (50,0)		50,0 (5,8–84,5)		1,00	-
2009	5 (17,9)	4 (80,0)		20,0 (0,8–58,2)		1,62 (0,30–8,90)	-
2010	6 (21,4)	3 (50,0)		50,0 (11,1–80,4)		0,72 (0,12–4,34)	-
2011	8 (28,6)	6 (75,0)		25,0 (3,7–55,8)		1,40 (0,28–6,96)	-
2012	5 (17,9)	2 (40,0)		60,0 (12,6–88,2)		0,65 (0,09–4,62)	-

S(t): probabilidade de sobrevivência em função do tempo; IC95%: intervalo de confiança de 95%; HR: razão de riscos proporcionais.

^a^ Valor de p do teste c^2^ (qui-quadrado).

^b^ Valor de p do teste Log-rank.

^c^ HR ajustada por faixa etária, escolaridade, raça, situação conjugal e extensão da doença.

^d^ HR ajustada por faixa etária, escolaridade, raça e extensão da doença.

### Bexiga (C67)

O câncer de bexiga teve 282 casos registrados (10,0% do total). Em cinco anos, 30,5% foram a óbito e a probabilidade de sobrevivência foi de 69,5% (IC95% 63,8–74,5) (Tabela 1 e Figura 1). Foi mais frequente em homens, com 70 anos ou mais, com nove anos ou mais de escolaridade, da raça branca, com companheiro e diagnóstico localizado. As variáveis associadas à sobrevivência foram faixa etária e extensão da doença. Na análise ajustada, foram incluídas as variáveis faixa etária, escolaridade, raça e extensão da doença. Apenas ter 70 anos ou mais foi fator independente para pior prognóstico, com aumento de seis vezes no risco de óbito (Tabela 4).

### Leucemia mieloide aguda (C92.0)

A LMA foi registrada em 28 casos e, desses, 60,7% foram a óbito. Em cinco anos, a probabilidade de sobrevivência foi de 39,3% (IC95% 21,7–56,5) (Tabela 1 e Figura 1). A distribuição dos casos foi similar entre sexo e idade, mais frequente naqueles com nove anos ou mais de escolaridade e de raça branca. Nenhuma das características foi associada à sobrevivência.

## DISCUSSÃO

Os achados da presente pesquisa demonstram que a sobrevivência e os fatores prognósticos variam de acordo com cada tipo de câncer relacionado ao tabaco no município de Florianópolis (SC). A maior probabilidade de sobrevivência foi encontrada no câncer de rim (75,7%), enquanto as menores, no câncer de pâncreas (14,3%), fígado (19,4%) e traqueia, brônquios e pulmão (22,8%). Os valores obtidos podem ser justificados por Santa Catarina apresentar longevidade 3,2 anos acima da média nacional e ter como perfil epidemiológico da população idosa a tripla carga de doenças, com forte predomínio das condições crônicas^13^. O Brasil está entre os países com maior redução na prevalência de tabagismo na população no período de 1990 a 2015, devido à adoção de políticas públicas^3^. Cabe destacar que há maior prevalência de adultos fumantes com 18 anos ou mais (14,7%) na região Sul, o que contribui como fator de risco para o aumento dessas doenças, e muitos dos fatores de risco para o óbito são relacionados ao tabagismo, como ser mais velho e ter menor escolaridade.

As neoplasias com maiores frequências encontradas neste estudo foram câncer de colo e reto, estômago e traqueia, brônquios e pulmão, o que se assemelha ao padrão encontrado nas regiões Sul e Sudeste que os coloca entre os cinco tipos mais incidentes. Os estudos que analisam a sobrevivência e os fatores prognósticos desses grupos de neoplasias são escassos. Visto que apresentam comportamentos diferentes, cada um dos tipos de câncer será discutido separadamente.

### Câncer de lábio e cavidade oral

Os achados deste estudo apresentam probabilidade de sobrevida de 57,9% e 65% para homens e mulheres, respectivamente. Outra pesquisa realizada no Rio Grande do Norte, que avaliou apenas dados sobre câncer de lábio, identificou que a sobrevida em cinco anos foi de 88,9% para homens e 87,3% para mulheres^14^. Pessoas com diagnóstico de cânceres de lábio, boca e faringe (CID-10 C00-C14) tiveram sobrevivência de 33,3% ao final de cinco anos de acompanhamento em Santa Catarina e o estadiamento ao diagnóstico foi o maior fator de risco para o óbito^15^. Entretanto, os casos de lábio e cavidade oral foram classificados no mesmo grupo. Nos dois estudos, a maioria dos casos foi diagnosticada no sexo masculino e em idade avançada, corroborando estes resultados. No estágio inicial, o câncer de lábio e cavidade oral costuma ser assintomático, o que leva ao diagnóstico tardio e com metástase^14,16^, piorando a sobrevivência e a qualidade de vida.

### Câncer de orofaringe

Pesquisas têm demostrado maior acometimento desse tipo de câncer no sexo masculino, na faixa etária entre 50–60 anos, e baixas taxas de sobrevivência, o que vem ao encontro dos achados deste estudo^17,18^.

Estudo realizado no Sul do Brasil com o intuito de observar a sobrevivência após câncer de orofaringe e boca identificou piores índices de sobrevida em indivíduos com tumores de orofaringe, associando tal dado à presença de metástase cervical e à maior disseminação tumoral, além da localização em regiões de difícil visualização e diagnóstico, o que contribui negativamente para as taxas de sobrevida^18^.

A literatura também indica que o abuso de álcool e a presença de doença em estágio avançado atuam como principais fatores de risco para piores resultados de sobrevida^17^.

### Câncer de esôfago

Uma revisão sistemática realizada em 2015 mostrou que a sobrevida por câncer de esôfago, em cinco anos, varia entre 10% e 15%, considerada baixa. Ainda, que é uma doença rara antes dos 30 anos de idade^19^. No presente estudo, a maior frequência foi na faixa etária entre 50 e 59 anos. Também foi realizado um levantamento com dados de diagnóstico entre os anos de 1997 e 2006 na Alemanha e nos Estados Unidos (EUA), no qual a sobrevida em cinco anos foi de 18% na Alemanha e de 17% nos EUA^20^, mais baixa do que a encontrada no presente estudo. A baixa probabilidade de sobrevivência ao câncer de esôfago é reflexo do diagnóstico tardio, que na maioria das vezes se deve à ausência de sinais clínicos no estágio inicial^21^, apesar de nossos resultados não apresentarem fatores de pior prognóstico.

### Câncer de estômago

A taxa de sobrevivência em cinco anos para câncer de estômago é em torno de 31%, dado encontrado em estudos realizados nos Estados Unidos e na Alemanha, o que condiz com os achados deste estudo^20,22^. Em Florianópolis, também foram encontradas taxas de sobrevida abaixo de 50% para ambos os sexos (45,1% para as mulheres e 36,1% para os homens)^23^.

Apesar dos avanços no diagnóstico e tratamento, a doença mantém-se com alta letalidade, o quinto câncer mais frequentemente diagnosticado e o terceiro mais letal. As taxas médias de sobrevivência refletem o fato de que a maioria das pessoas é diagnosticada com presença de metástases. Trata-se de uma doença com heterogeneidade histológica e etiológica^22^; portanto, a prevenção primária através do controle de fatores de risco bem estabelecidos (como fumo, consumo excessivo de álcool, ingesta de alimentos com alto teor de sal) é essencial para a redução das taxas de incidência, e alertas sobre os sinais e sintomas são essenciais para o diagnóstico precoce.

### Câncer de colón e reto

Os achados do presente estudo vão ao encontro de pesquisas que identificaram taxa de sobrevida global, em cinco anos, para pessoas com câncer colorretal, entre 63 a 65% e pior prognóstico na faixa etária acima de 70 anos^23,24^.

As taxas de sobrevida podem variar de acordo com o estágio e a localização primária da doença. Nas neoplasias metastáticas ou estágio IV, a taxa de sobrevida relativa é baixa, o que também foi identificado neste estudo. Logo, os tumores em cenário metastático tornam o tratamento mais complexo e tendem a ter pior perspectiva^25^. Além disso, destaca-se que a maior sobrevivência pode estar relacionada ao início precoce do tratamento, independentemente de outras características^24^.

### Câncer de fígado

Apesar de algumas melhorias nas taxas de sobrevida do câncer de fígado nas décadas mais recentes, o prognóstico geral para tal doença permanece desfavorável. Estudo realizado nos Estado Unidos, em 2017, revela taxa de sobrevida em cinco anos de 21% no geral, variando de 4% para doença em estágio distante a 37% para doença localizada^26^, o que se aproxima do dado encontrado neste estudo.

A literatura denota maiores taxas quanto à sobrevida desses indivíduos, o que pode estar relacionado a melhor controle dos fatores de risco, detecção e tratamento precoces, remetendo à qualidade da assistência em saúde prestada^24^.

### Câncer de pâncreas

O câncer de pâncreas apresenta uma alta letalidade e apesar de não estar entre os dez tipos mais comuns, representa a oitava principal causa de morte. Em grande parte de seus casos, o diagnóstico é realizado em fase tardia, o que dificulta a possibilidade de tratamento curativo. A taxa de sobrevida conhecida é por volta de 5% em cinco anos^28^. No presente estudo, a taxa de sobrevida foi levemente superior, de 12,9% para mulheres e 16,0% para homens, o que ainda reflete uma baixa sobrevida.

Quanto aos fatores de risco independente para o óbito, apenas a idade avançada foi significativa para esses achados, o que encontra sustentação na literatura em relação ao predomínio de indivíduos acometidos na sexta década de vida^28,29^.

### Câncer de brônquios, traqueia e pulmão

Neste estudo, o câncer de brônquios, traqueia e pulmão foi o segundo mais diagnosticado. No entanto, no mundo, este é o tipo mais incidente, com 2,1 milhões de casos, acometendo mais os homens e geralmente detectado em casos mais avançados^4^.

Pesquisa realizada em Florianópolis apresentou resultados similares aos aqui encontrados, com taxas de sobrevivência de 22,5%^23^. Tais taxas são superiores às relatadas em levantamento realizado na Espanha, que em cinco anos, obteve 14%^30^.

O risco de ocorrência do câncer de pulmão e consequente morte pela doença está relacionado a maior intensidade de exposição ao tabagismo, além de fatores como a exposição ocupacional a agentes químicos ou físicos^30^.

### Câncer de bexiga

O câncer de bexiga é o mais frequente no trato urinário e acomete com mais frequência homens, resultados similares aos deste estudo^4^. A sobrevida global em cinco anos identificada neste estudo está entre os 60% a 80% descritos na literatura^23,31^.

Entre os casos recém-diagnosticados, aproximadamente 30% têm se apresentado com a doença localmente avançada ou metastática. No entanto, o maior acesso da população a métodos de diagnóstico por imagem tem ampliado os diagnósticos precoces, cujas taxas refletem resultados muito mais favoráveis^31^.

### Leucemia mieloide aguda

A LMA é um dos quatro tipos mais frequentes de leucemia e o que mais acomete adultos^4^. Os achados deste estudo são semelhantes aos encontrados em pesquisa realizada nos Estados Unidos, que demonstrou que indivíduos com diagnóstico de LMA e 75 anos ou mais tiveram taxas de sobrevida mais baixas em comparação com outras faixas etárias^32^.

Outro estudo realizado nos EUA denota que idosos com LMA têm mais comorbidades e maiores taxas de complicações quando comparados a controles sem câncer. Assim, a presença de comorbidades, disfunção orgânica anterior e uma percepção de inaptidão à quimioterapia intensiva interferem nas taxas de sobrevida desta população^33^.

## CONSIDERAÇÕES FINAIS

Este estudo apresentou a taxa de sobrevivência e fatores prognósticos para 13 tipos de câncer que são relacionados ao tabaco. Os fatores de pior prognóstico e as taxas de sobrevivência diferem entre os tipos. O estadiamento foi o fator de risco de pior prognóstico mais frequente, demonstrando a importância de campanhas para alertar sobre prevenção primária e diagnóstico precoce. O tabagismo permanece como um dos principais fatores de risco para diversas DCNTs, entre elas o câncer, e com isso impacta diretamente na carga de doenças, com custos sociais, econômicos e perda da qualidade de vida.

O estudo apresenta limitações relacionadas à utilização de dados secundários, o que resulta em ausência de informações que poderiam complementá-lo, como dados sobre o perfil socioeconômico e status do tabagismo. Da mesma forma, a ocorrência de dados incompletos devido à falta da informação ou erro de digitação no SIM dificulta a análise dos dados e pode interferir nos resultados da pesquisa. Destaca-se também a falta de informação sobre o hábito tabágico durante a vida, já que esta poderia reforçar para a população a importância do tabagismo enquanto fator de risco para o desenvolvimento e evolução da doença.

Entre os pontos positivos, destaca-se os índices de qualidade do RCBP, o percentual de verificação histológica – 92,6% –, o percentual de casos identificados somente pela declaração de óbito – 2,68% – e o percentual de casos diagnosticados com localização primária desconhecida (C80.9) – 0,83% – durante o período de 2008 a 2012. Além disso, o SIM de Santa Catarina tem alta completude.

Florianópolis segue o comportamento do Sul do país, como já identificado em outros estudos, tal como o panorama internacional para alguns tipos de câncer. A Política Nacional para a Prevenção e Controle do Câncer baseia-se em dados de estudos nacionais e internacionais para o direcionamento de suas ações. Nesse sentido, este estudo pode contribuir para a adequação das políticas e ações às realidades locais, visto que existe uma diversidade sociocultural, econômica e populacional muito grande no país. Tais realidades impactam diretamente nos dados encontrados nas diferentes regiões brasileiras. Para o controle do câncer, é essencial conhecer as características locais, que dialogam com as características sociais e de acesso à saúde do território. Compreender o comportamento da doença através da análise de sobrevida é uma das formas de embasar a realização dos trabalhos de prevenção, promoção e conscientização.

Os achados deste estudo ainda reforçam a necessidade de investimento público na política de controle do tabagismo, a fim de ampliar e garantir o acesso da população às políticas a nível local, visto que todas as neoplasias aqui abordadas têm relação com o uso do tabaco e integram os planos de enfrentamento à DCNT. Apesar das leis que respaldam os casos de suspeita ou diagnóstico de neoplasia, da lei antitabaco do município de Florianópolis e da Política de Controle do Tabagismo, é necessário extrapolar os dados encontrados para os profissionais de saúde e gestores, visando o fortalecimento e consolidação das medidas de prevenção, detecção precoce, rastreamento e tratamento adequado para o câncer.
